# Cysteine-Rich Atrial Secretory Protein from the Snail *Achatina achatina*: Purification and Structural Characterization

**DOI:** 10.1371/journal.pone.0138787

**Published:** 2015-10-07

**Authors:** Sergey Shabelnikov, Artem Kiselev

**Affiliations:** 1 Department of Cytology and Histology, Saint-Petersburg State University, St. Petersburg, Russia; 2 Laboratory of Cell Morphology, Institute of Cytology, Russian Academy of Sciences, St. Petersburg, Russia; 3 Institute of Molecular Biology and Genetics, Almazov Federal Medical Research Centre, St. Petersburg, Russia; Centro Nacional de Biotecnologia - CSIC, SPAIN

## Abstract

Despite extensive studies of cardiac bioactive peptides and their functions in molluscs, soluble proteins expressed in the heart and secreted into the circulation have not yet been reported. In this study, we describe an 18.1-kDa, cysteine-rich atrial secretory protein (CRASP) isolated from the terrestrial snail *Achatina achatina* that has no detectable sequence similarity to any known protein or nucleotide sequence. CRASP is an acidic, 158-residue, N-glycosylated protein composed of eight alpha-helical segments stabilized with five disulphide bonds. A combination of fold recognition algorithms and *ab initio* folding predicted that CRASP adopts an all-alpha, right-handed superhelical fold. CRASP is most strongly expressed in the atrium in secretory atrial granular cells, and substantial amounts of CRASP are released from the heart upon nerve stimulation. CRASP is detected in the haemolymph of intact animals at nanomolar concentrations. CRASP is the first secretory protein expressed in molluscan atrium to be reported. We propose that CRASP is an example of a taxonomically restricted gene that might be responsible for adaptations specific for terrestrial pulmonates.

## Introduction

Gastropods are the largest and most diverse group of molluscs, with about 100,000 species inhabiting marine, freshwater and terrestrial habitats [1]. The gastropod heart shares striking similarity with the vertebrate heart: it possesses a pericardial sac, a chambered structure, valves, trabeculae and myogenic rhythm [2]. The relatively simple organization of the nervous and cardiovascular systems has made gastropods popular and important animal models in neurobiology research.

Although the gastropod heart is primarily a blood-pumping and ultrafiltration organ, Cottrell and Osborne [3] identified a neurohaemal area on the inner surface of the *Helix pomatia* atrium. Numerous cardioactive peptides have since been identified, as reviewed in [4]. These include the ~8.9-kDa sodium influx-stimulating peptide (SIS) detected in the neurohaemal areas and pericardium of *Lymnaea stagnalis* [5] and the ~7-kDa large cardioactive peptide (LCP) in *Helix aspersa*. LCP is the highest molecular mass neuropeptide known to be released from the atrium into the haemolymph. LCP regulates the heart, gut and neuromuscular activity [6]. However, thus far, all peptides identified in the molluscan heart are of neuronal origin.

Granular cells, an interesting feature of the gastropod heart, are specialized secretory cells attached to the myocardial surface of the atrium. Until recently, they had only been described in *Pulmonates*: *L*. *stagnalis* [7], *H*. *pomatia* [8], *H*. *aspersa*, *Strophocheilus oblongus* [9], *Achatina fulica* [10] and *Achatina achatina* [11]. The secretory granules of granular cells have been immunostained with antibodies against atrial natriuretic peptide [12], Hsp70 [10], substance P and FMRFamide [13], and serotonin and histamine [11]. Granule exocytosis in granular cells has been studied in detail [11], and it has been proposed that snail atrial granular cells are functionally analogous to vertebrate mast cells [11,14]. Atrial granular cells form close contacts with nerve terminals and undergo total degranulation following stimulation of the heart nerve [14].

Our study was motivated by the observation that granular cells release proteins into the heart lumen upon stimulation [14]. We purified, cloned and characterized the most abundant protein released into the haemolymph from the *A*. *achatina* atrium. We called the ~16-kDa protein ‘cysteine-rich atrial secretory protein’ (CRASP) because it contains ten cysteine residues and its expression is highest in the atrium. This is the first report to describe the isolation and characterization of a secretory protein expressed in the atrium of gastropod molluscs.

## Results

### Purification of CRASP

**Fig 1:**
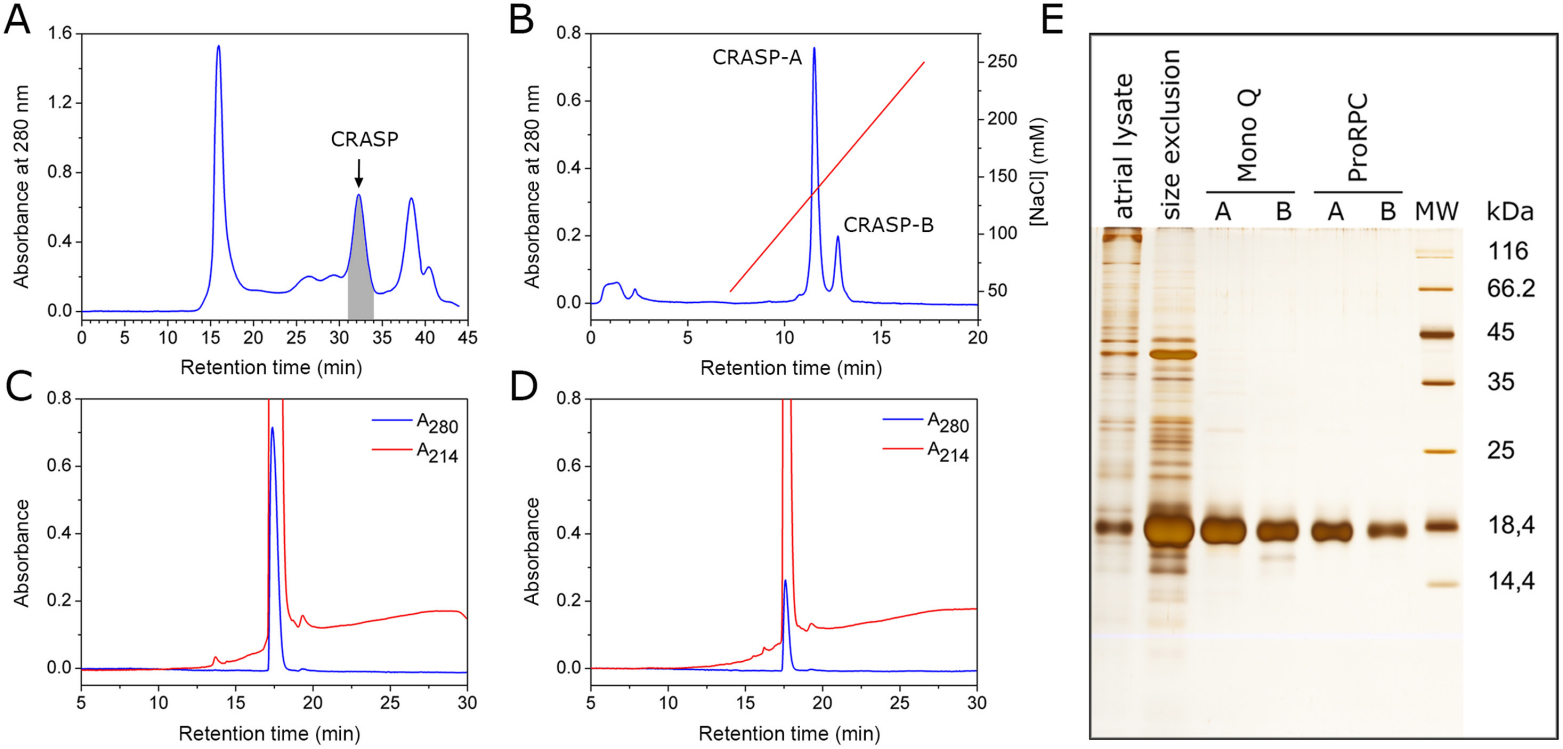
Purification of CRASP isoforms from the atria of *A*. *achatina*. (A) Semi-preparative size-exclusion chromatography on a Superdex 200 column. Fraction from 31 to 34 min (shaded grey) was collected and subjected to anion exchange chromatography. (B) Separation of isoforms on a Mono-Q anion exchange column. Fractions from the two major peaks were designated CRASP-A and CRASP-B. Final purification of CRASP-A (C) and CRASP-B (D) on a ProRPC C4 reversed phase column. (E) All fractions were analysed with SDS-PAGE (15% gel) and silver staining.

CRASP was isolated from the atria of *A*. *achatina* snails through a combination of size-exclusion, anion exchange and reversed phase chromatography (Fig 1). In the first purification step, CRASP was obtained in a single peak with a retention time of about 32 min (Fig 1A). Subsequent anion exchange purification yielded two peaks with the same mobility on SDS-PAGE (Fig 1B and 1E), indicating the presence of two distinct isoforms. Pooled fractions from these peaks, were designated CRASP-A and CRASP-B, eluted at ~136 mM and ~160 mM NaCl, respectively. The native proteins fractions were used for analytical isoelectric focusing and structural studies in size-exclusion chromatography and CD spectroscopy. After a final reversed phase HPLC purification, we obtained virtually homogeneous samples of CRASP-A (Fig 1C and 1E) and CRASP-B (Fig 1D and 1E), which were used for Edman degradation and mass spectrometry.

### cDNA cloning and sequence analysis

**Fig 2:**
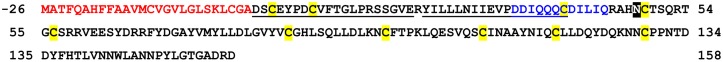
Deduced amino acid sequence of CRASP. The signal peptide is shown in red, a low compositional complexity region is shown in blue, cysteines are shaded yellow, and a potential N-glycosylation site is shaded black. The N-terminal and internal peptide sequences detected by Edman degradation are underlined.

Purified CRASP was subjected to automated Edman degradation, and the sequence of the 20 N-terminal residues was obtained. The sequence of a 19-residue-long internal peptide was obtained by digestion of the reduced and alkylated protein with ArgC peptidase and subsequent Edman degradation (Fig 2). The partial sequence information was used to design degenerate primers and to amplify full-length CRASP cDNA through 3′ and 5′ rapid amplification of cDNA ends. The CRASP cDNA was 882 bp, comprising a 5′-untranslated sequence of 63 bp, an open reading frame (ORF) of 552 bp and a 3′-untranslated region of 271 bp. The first ATG at position 64–66 was assigned as the start codon. A polyadenylation signal (AATAAA) was found 15 bp upstream of the poly(A)+ tail. The ORF encoded a protein of 184 amino acid residues (Fig 2; accession number W1I921 in UniProtKB). A 26-residue signal peptide precedes the N-terminal Asp residue identified by Edman degradation. The mature protein comprises 158 amino acid residues with a theoretical molecular mass of 18120.3 Da. A region of low compositional complexity was detected at position Asp33-Gln34. One potential N-glycosylation site was predicted at Asn48. The mature protein contains 21 negatively charged residues (Asp + Glu) and 12 positively charged residues (Arg + Lys), giving a calculated pI of 4.57.

### Novelty of CRASP

An initial BLASTP search against the GenBank and UniProtKB non-redundant protein databases using the CRASP sequence returned no hits with an E-value better than 0.01. More sensitive tools (CS-BLAST, HMMER and HHblits) also failed to find any significant matches. Remarkably, TBLASTN searches in the genomes of *Lottia gigantea*, *Aplysia californica* and *Biomphalaria glabrata*, the only gastropods with sequenced genomes, did not identify any discernable homologs. Similarly, no homologs were identified in the transcriptome databases of sea slugs (*A*. *californica*, *Tritonia diomedea* and *Placobranchus ocellatus*) and freshwater pulmonate snails (*L*. *stagnalis* and *B*. *glabrata*). These results indicate that CRASP is novel and taxonomically restricted.

### Mass, charge, size and shape

**Fig 3:**
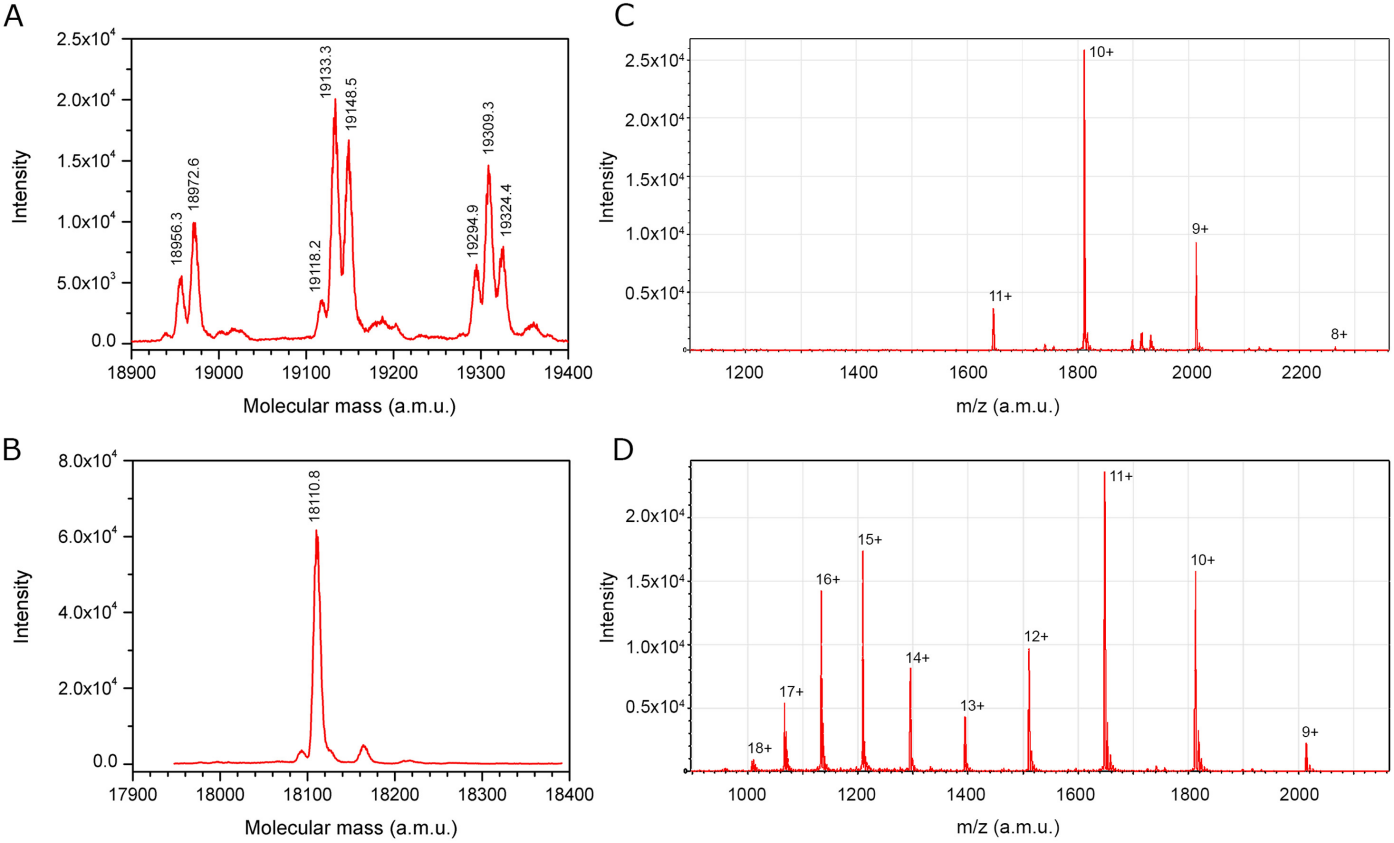
ESI MS analysis of CRASP in water/acetonitrile with 0.25% formic acid. (A) A typical deconvoluted ESI mass spectrum of CRASP containing N-linked glycan. Note the multiple peaks due to glycan heterogeneity. (B) The deconvoluted ESI mass spectrum of the deglycosylated protein showed a single peak of 18110.8 Da. ESI mass spectra of deglycosylated, non-reduced CRASP (C) and CRASP reduced with dithiothreitol (D). Peaks are labelled according to the charge states of the protein ion.

Analysis of the purified CRASP isoforms with electrospray ionization mass spectrometry (ESI MS) revealed clear heterogeneity: three clusters of peaks differed by about 161 ± 1 Da, and the three peaks in each cluster differed by 15 ± 1 Da (Fig 3A). Treatment of CRASP with N-glycosidase F resulted in the disappearance of heterogeneity and the appearance of a single peak with a reduced molecular mass of 18110.8 Da (Fig 3B). These data suggested that CRASP was N-glycosylated. The observed mass change ranged from 845.5 Da to 1213.6 Da. Thus, CRASP is modified by addition of several hexose residues (162 Da) to an N-linked core glycan.

The unfolding of deglycosylated CRASP was monitored with LC-ESI MS in water/acetonitrile with 0.25% formic acid. The reductive cleavage of disulphide bonds by dithiothreitol led to significant changes in the ESI charge state distribution. The spectrum of non-reduced CRASP had a narrow charge state distribution with a maximum at 10^+^ (Fig 3C) The unfolded form of the protein showed a much broader, bimodal charge state distribution with maxima at 11^+^ and 15^+^ (Fig 3D). The observed maxima correlate with the number of basic amino acid residues: nine Arg, three Lys and three His.

The two CRASP isoforms were not distinguished by the ESI MS but were easily separated by anion exchange chromatography, indicating that their net charge differed. Analytical isoelectric focusing showed that CRASP-A and CRASP-B had isoelectric points of 4.37 and 4.30, respectively. Thus, CRASP is an acidic protein, negatively charged at neutral pH.

**Fig 4:**
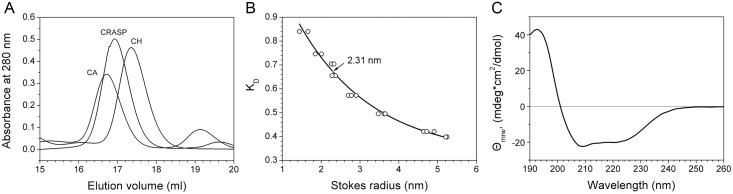
Biophysical characterization of CRASP. (A) Size-exclusion chromatography showed that deglycosylated CRASP migrated unusually fast compared to carbonic anhydrase (CA) and chymotrypsinogen (CH). (B) Determination of the Stokes radius of the deglycosylated protein by size-exclusion chromatography. The elution position (arrow) and estimated Stokes radius are indicated. (C) The CD spectrum showed a strong maximum at 192 nm and two minima at 208 nm and 222 nm, the characteristic spectral features of an *α*-helical conformation. Only the CD spectrum of isoform A is shown because the spectra of the isoforms were almost identical.

We next examined the hydrodynamic properties of CRASP using size-exclusion chromatography. The deglycosylated protein migrated unusually fast for an 18.1 kDa protein on a size-exclusion column and had an elution volume of 16.92 ml (Fig 4A). The protein with intact glycan migrated even faster (S1 Fig). This anomaly can be explained by assuming that the CRASP molecule is slightly elongated. We estimated that CRASP has a Stokes radius of 2.31 nm (Fig 4B), compared to the 1.73-nm minimal radius of a spherical protein of the same mass. In agreement with a slightly elongated shape, CRASP had a frictional ratio of 1.3, whereas the globular proteins carbonic anhydrase and chymotrypsinogen had frictional ratios close to 1.2 (S2 Table).

### Secondary structure of CRASP

For optimal CD spectra, native CRASP isoforms obtained through an anion exchange purification step were rechromatographed on a size-exclusion column in a low-UV-absorbing buffer (see Materials and Methods). The proteins ran with the same retention times of 32.7 min (S1 Fig). This value is close to the retention time observed for the CRASP-containing peak in the first purification step, indicating preservation of the compact native state of the protein in the course of isolation. Further, we investigated the secondary structure of CRASP using CD spectroscopy in the far UV region (Fig 4C). The CD spectra showed a strong positive peak at 192 nm and two negative peaks at 208 nm and 222 nm, the characteristic spectral features of an *α*-helical conformation [15]. The normalized standard deviation of 0.01 between the CD spectra of the two isoforms indicated nearly identical secondary structure content. Therefore, it is very likely that CRASP adopts a structure consisting predominantly of *α*-helices.

To characterize the secondary structure further, the CD spectra of the isoforms were analysed using four different deconvolution algorithms: SELCON3, CONTIN/LL, CDSSTR and K2D3 (Table 1). The results obtained with the different methods are in good agreement. The following average proportions of secondary structure elements were obtained: 60% *α*-helix, 5% *β*-strand, 11% *β*-turn and 24% unordered residues. The number and average length of the *α*-helical and *β*-strand segments were estimated from the CD spectra: CRASP consists of eight *α*-helices with an average length of 12 residues and two *β*-strands with an average length of four residues.

**Table 1:** CD analysis of CRASP secondary structure. The proportions of the *α*-helix (*α*), *β*-strand (*β*), *β*-turn (*T*) and unordered (*U*) secondary structure conformations from each analysis are shown. The secondary structure fractions (*f*), the number of residues (*n*) and the number (*N*) and average length (*L*) of the secondary structure segments are also shown. Note the good agreement across results obtained with different algorithms. The average of the calculations across SELCON3, CONTIN/LL and CDSSTR is shown in bold-face type.

Method	*α*-Helix				*β*-Strand				*β*-Turn		Unordered	
	*fα*	*nα*	*Nα*	*Lα*	*fβ*	*nβ*	*Nβ*	*Lβ*	*fT*	*nT*	*fU*	*nU*
SELCON3	0.591	93.3	7.8	12.0	0.051	8.0	2.1	3.8	0.108	17.0	0.241	38.1
CONTIN/LL	0.576	90.9	7.6	12.0	0.056	8.8	2.2	4.0	0.115	18.1	0.255	40.2
CDSSTR	0.615	97.2	7.9	12.3	0.045	7.1	1.6	4.5	0.106	16.6	0.230	36.3
**Average**	**0.594**	**93.8**	**7.8**	**12.1**	**0.051**	**7.8**	**2.0**	**4.1**	**0.109**	**17.0**	**0.242**	**38.2**
K2D3	0.602	95.1			0.044	6.9						

**Fig 5:**
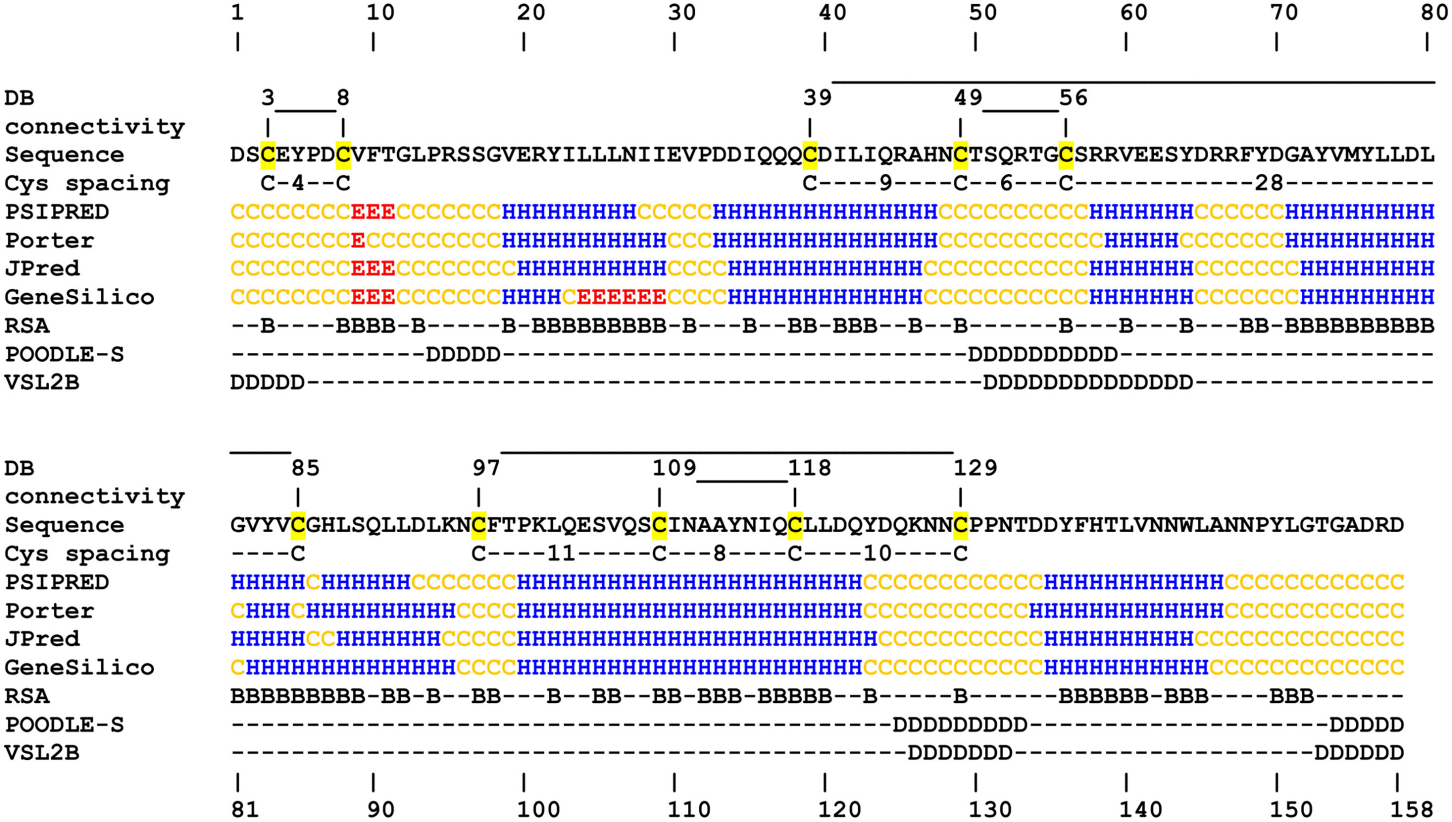
Prediction of secondary structure, solvent accessibility and disorder. The disulphide bonding pattern was determined experimentally. Cysteine spacing motifs are shown; the cysteine residues are shaded yellow. The secondary structure was predicted using PSIPRED, Porter, JPred and GeneSilico. Relative solvent accessibility was predicted using Spine-X. Short disordered regions were predicted using POODLE-S and VSL2B. ‘H’, ‘E’ and ‘C’ denote α-helices, β-strands and coils, respectively. ‘B’ denotes buried residues (relative solvent accessibility <25%), and ‘D’ denotes predicted disordered residues.

Next, we applied computational tools to predict the secondary structure, relative solvent accessibility and disorder (Fig 5). Four different approaches predicted the positions of the secondary structure elements in the CRASP sequence with good general agreement. Data averaging showed that the majority of the protein adopted an *α*-helical conformation (~54%) with only a minor amount of *β*-structure (~3%). The consensus structure predicted seven *α*-helices with two N-terminal *β*-strands. Prediction of the relative solvent accessibility revealed a stretch of 19 buried residues at position Gly71-Ser89. The region is probably involved in the formation of a hydrophobic core. Stretches with a high disorder tendency were confidently assigned to two loops and the C-terminus of the protein.

### Determination of the disulphide bonding pattern

The calculated molecular mass of the mature deglycosylated protein (18121.2 Da, assuming one glycosylation site) is larger than that of deglycosylated CRASP as determined by ESI MS (18110.8 Da). The presence of five disulfide bonds accounts for the observed difference of 10.4 Da. Indeed, after reduction of CRASP with dithiothreitol, the observed molecular mass changed to 18120.7 Da. After reduction and alkylation with iodoacetamide, ESI MS showed the incorporation of 10 carboxamidomethyl groups (18690.6 Da, average mass change of +570 Da). No incorporation was observed when the reduction step was omitted, suggesting that CRASP contains 10 cysteine residues involved in the formation of five disulphide bonds.

The arrangement of the disulphide bonds was established by ESI MS analysis of the trypsin-digested, deglycosylated, non-reduced protein isoforms. Table 2 summarizes the molecular masses of four disulphide-bonded peptides and six fragments obtained upon reduction. Although the peptides AHNCTSQR and TGCSR were not observed, the fifth disulphide bond was assigned to Cys49 and Cys56 by elimination, because there are no free cysteine residues in CRASP. Thus, five disulphide bonds were identified in CRASP, an N-terminal simple bond Cys3-Cys8 and two pairs of enclosed bonds: Cys39-Cys85/Cys49-Cys56 and Cys97-Cys129/Cys109-Cys118 (Fig 5). The first and second pairs of enclosed bonds showed cysteine spacing motifs Cys-9-Cys-6-Cys-28-Cys and Cys-11-Cys-8-Cys-10-Cys, respectively.

**Table 2:** ESI MS analysis of CRASP tryptic peptides containing disulphide bonds. Note the monoisotopic mass change of +0.97 Da in the peptide obtained from isoform B, shown in bold-face type.

Peptide sequence	Cys pairing	Observed charges	Monoisotopic mass (a.m.u.)		
			Experimental		Calculated
			CRASP-A	CRASP-B	
|¯¯¯¯| DS**C**EYPD**C**VFTGLPR	Cys3-Cys8	1+, 2+	1698.72	1698.69	1698.70
YILLLNIIEVPDDIQQQ**C**DILIQR FYDGAYVMYLLDLGVYV**C**GHLSQLLDLK	Cys39-Cys85	4+, 5+	6072.97	6073.00	6073.14
|¯¯¯¯¯¯¯| N**C**FTPK NN**C**PPNTDDYFHTLVNNWLANNPYLGTGADR	Cys97-Cys129	3+, 4+	4211.77	4211.73	4211.89
|¯¯¯¯¯¯¯¯| LQESVQS**C**INAAYNIQ**C**LLDQYDQK	Cys109-Cys118	2+, 3+	2884.30	**2885.27**	2884.33
DS**C**EYPD**C**VFTGLPR	reduced	1+, 2+	1700.72	1700.72	1700.72
YILLLNIIEVPDDIQQQ**C**DILIQR	reduced	2+, 3+	2867.58	2867.59	2867.55
FYDGAYVMYLLDLGVYV**C**GHLSQLLDLK	reduced	2+, 3+	3207.60	3207.63	3207.60
N**C**FTPK	reduced	1+	708.352	708.353	708.326
NN**C**PPNTDDYFHTLVNNWLANNPYLGTGADR	reduced	2+, 3+, 4+	3505.55	3505.53	3505.57
LQESVQS**C**INAAYNIQCLLDQYDQK	reduced	2+, 3+	2886.33	**2887.30**	2886.35

ESI MS analysis of tryptic peptides detected a monoisotopic mass change of +0.97 Da in the peptide LQESVQSCINAAYNIQCLLDQYDQK, derived from the B isoform. The observed mass change matched closely with those resulting from deamidation of Asn to Asp or Gln to Glu (+0.98 Da).

### Assigning a fold to CRASP

The CRASP sequence was submitted to 15 different fold-recognition programs. Among the pool of 145 identified templates, 82 hits belonged to the all-alpha SCOP structural class, 26 hits to alpha + beta, 24 hits to alpha/beta, and 13 hits to all-beta, *g* and *f* structural classes. Because CRASP has been experimentally characterized as an all-alpha protein, the following analysis was restricted to the all-alpha templates. The CRASP sequence was classified as a ‘hard target’ for comparative modelling with the LOMETS meta-threading server, indicating that no statistically significant template hit was detected with the current threading methods. At a low confidence score, the ranking of templates was close to random. However, templates with a correct fold can still be present among the top 10 hits.

Threading templates were sorted based on their distance from the top QUARK *ab initio* model, with the intent to detect templates with the correct fold, assuming that a match between the real structure and the *ab initio* folding model is significant and often indicates the correctness of the fold [16,17]. As a distance measure, we used the TM-score and the FATCAT p-value because these are distinct approaches to measuring structural similarity.

**Fig 6:**
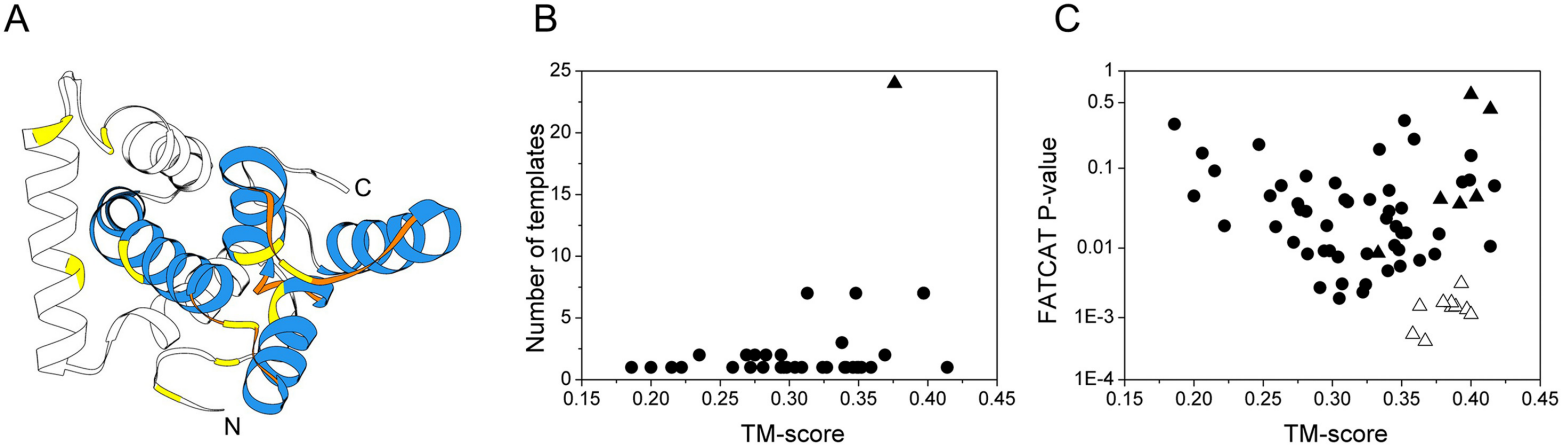
The use of *ab initio* folding simulation to detect correct templates from a pool of structures identified by threading methods. (A) Top QUARK *ab initio* model refined with ModRefiner. The Cα-Cα′ distance restraints used to direct folding are satisfied (yellow). The C-terminal part of the protein shows four α-helices (blue) with right-handed superhelical topology. (B) Templates sorted according to an average TM-score calculated on the fold level, revealing SCOP a.118, an all-alpha right-handed superhelical fold (triangle). (C) Templates sorted according to the TM-score and the FATCAT p-value. A cluster of SCOP a.118.9 superfamily templates is evident (open triangles). Other templates from the SCOP a.118 fold (filled triangles) and other all-alpha templates (filled circles) are also shown.

**Fig 7:**
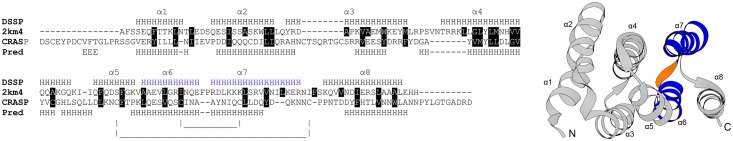
Sequence-structure alignment of the CRASP sequence with the all-alpha, right-handed superhelical fold of the 2KM4 template. The representative original threading alignment produced by the SP3 program (left panel) and the 3D structure of the 2KM4 template (right panel) are shown. The secondary structure is labelled ‘H’ for *α*-helix and ‘E’ for *β*-strand, where ‘DSSP’ and ‘Pred’ denote the assignment by DSSP and the prediction by PSIPRED, respectively. Alpha helices *α*1-*α*8 are shown. Residues involved in hydrophobic core packing in the experimental structure are shaded black. Note the good match in hydrophobicity and the match of the *α*-helical hairpin (blue), formed by the template’s sixth and seventh helices, with a long helical segment constrained by two disulphide bonds, which probably forms a similar super-secondary motif. The sequence identity is 11%.

The top model generated by QUARK is shown in Fig 6A (accession number PM0079929 in the Protein Model DataBase). The model had a seven-helix complex topology. Distance restraints derived from the disulphide bonding pattern were satisfied. Fig 6B shows the number of all-alpha templates grouped by SCOP fold level and plotted versus the average TM-score. The highest number of templates (24 hits) belonged to SCOP a.118, an all-alpha right-handed superhelical fold, with an average TM-score of 0.376. In Fig 6C, we plotted the FATCAT p-values of all-alpha templates versus the TM-scores. After sorting by the QUARK model, we found that the SCOP a.118.9 ENTH/VHS superfamily was clearly separated from the other all-alpha templates. The PDB IDs of these templates are **1DVP**, **1ELK**, **3LDZ**, **1X5B, 3CLJ**, **2DIW**, **1SZA**, **1SZ9**, **2KM4** and **4FLB**. The best match between the QUARK model and these templates was a group of four helices arranged in a right-handed superhelical fashion in the C-terminal region (Fig 6A). A representative threading alignment is shown in Fig 7. Remarkably, an alpha-hairpin formed by the sixth and seventh helices of the template matched to a long helix of CRASP that should form a similar super-secondary motif constrained by two enclosed disulphide bonds. This structural feature was recognized by threading programs and modelled by QUARK, allowing the assignment of the most favorable fold to CRASP.

### Tissue specificity of CRASP gene expression

A quantitative real-time PCR (qRT-PCR) analysis was performed to examine the tissue distribution of CRASP mRNA expression in adult *Achatina*. Because sequence data are scarce for this snail, we used orthologues of *α*-tubulin and 60S acidic ribosomal protein P0 (60SARP) genes from the closest relatives with available transcriptomes. The melting curves for all analysed genes exhibited single peaks, confirming the specificity of the amplification (S2 Fig). Agarose gel electrophoresis showed a single band of the expected relative mobility for each amplified gene (S3 Fig). The raw *Cq* values ranged from 25.2 to 28.2 for *α*-tubulin and from 21.2 to 24.3 for 60SARP. The median of the *Cq* distribution for *α*-tubulin and 60SARP was 26.5 and 22.3, respectively. The distribution of the *Cq* values is shown in S4 Fig.

**Fig 8:**
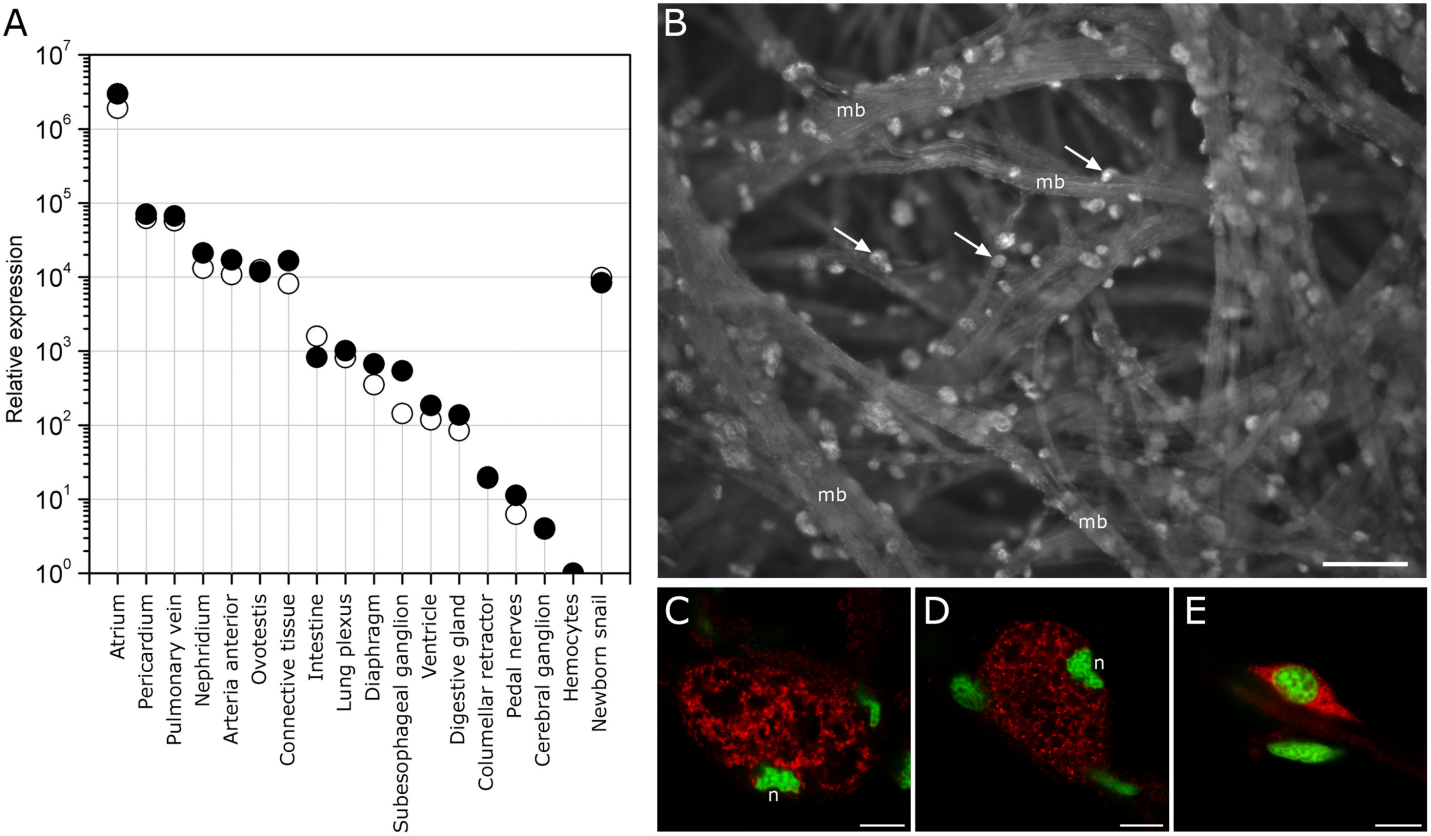
Tissue-specific expression of CRASP mRNA. (A) qRT-PCR analysis of CRASP mRNA expression in different tissues. Relative expression was calculated using *α*-tubulin (open circles) and 60S acidic ribosomal protein P0 (filled circles) as reference genes. A log scale (y-axis) was used. Data are presented as the mean of two replicate qRT-PCR reactions from pooled tissues from two snails. (B) FISH detection of CRASP mRNA in whole-mount atrium. The epifluorescent image demonstrates the distribution of transcripts within cells (arrows) located on the surface of muscle bundles (mb). The scale bar indicates 50 μm. (C, D) Confocal sections demonstrate the localization of transcripts in the intergranular space of the atrial granular cells. Note the specific eccentric location and irregular shape of the nucleus (n) in mature granular cells. (E) Rarely, transcripts were detected in small spindle-like cells. Scale bars indicate 7.5 μm. The hybrids were detected with avidin-Cy3 (red pseudo colour). DAPI was used as a general DNA dye (green pseudo colour).

Expression of CRASP mRNA was detected at different levels in a wide range of tissues (Fig 8A). The raw *Cq* values varied from 13 in the atrium to 36.6 in the haemocytes. The highest levels were detected in the atrium, where expression was 2.4 × 10^6^ times greater than in the haemocytes, which had the lowest levels. Relatively high expression was observed in the pericardium and pulmonary vein, but it was about 40-fold lower than expression in the atrium. The nephridium, arteria anterior, ovotestis and connective tissue showed medium levels of expression, about 180-fold lower than in the atrium. Low expression was observed in the other investigated tissues, at levels 2 × 10^3^-fold lower than in the atrium. Despite the proximity of the atrium and ventricle, the latter showed 16 × 10^3^-fold lower expression. The expression in whole newborn snail was 270-fold lower than in the atrium of the adult snail.

The localization of CRASP mRNA in the atrium was examined with fluorescent in situ hybridization (FISH). Large labelled cells were observed on the surface of muscle bundles (Fig 8B). These cells were clearly identified as granular cells based on the following morphological features: spheroid shape, size of ~30 μm, granular content and an eccentrically located and distorted nucleus. Confocal microscopy revealed a hybridization signal localized in the cytoplasmic space between tightly packed granules (Fig 8C and 8D). Rarely, a hybridization signal was detected in the cytoplasm of small spindle-like cells with a high nuclear-cytoplasmic ratio (Fig 8E). Negative controls showed no hybridization signal (data not shown).

### Stimulated secretion of CRASP

**Fig 9:**
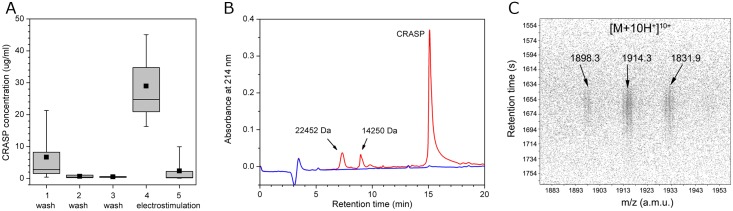
CRASP is secreted by the heart and has low abundance in the haemolymph. (A) Secretion of CRASP by the isolated heart. The protein concentration decreased significantly from the first wash to the third wash and increased in the fourth fraction collected upon electrostimulation of the heart nerve. Note the depletion of CRASP after the fourth fraction with continued stimulation. The mean (squares), median (lines), 25th to 75th percentiles (boxes) and range (whiskers) for six samples are shown. (B) Representative chromatograms of the third wash fraction (blue line) and the fraction collected upon stimulation (red line). Note that in addition to CRASP, two minor proteins are present. (C) LC-ESI MS identification of CRASP in the haemolymph. The fragment of the LC-MS 2D map shows a 10-fold charged ion species with typical heterogeneity.

To examine whether CRASP was released into the heart lumen, we applied a perfusion method to isolated hearts with electrostimulation of the heart nerve. Six independent experiments were performed, and the collected perfusion samples were analysed by HPLC. Pre-stimulation washings resulted in a gradual decrease in CRASP from 6.7 ± 7.7 μg/ml to 0.5 ± 0.2 μg/ml, while electrostimulation induced an increase to 29.0 ± 10.4 μg/ml (Fig 9A). Subsequent stimulation showed a decrease in CRASP to 2.4 ± 3.8 μg/ml, indicating rapid depletion of the protein. The total amount of CRASP released was 39.1 ± 15.4 μg per heart, which represents 1.2 ± 0.6% of the atrium dry weight. Moreover, we noted that CRASP was the most abundant protein released upon stimulation (Fig 9B). We only detected two other minor proteins of 14250 Da and 22452 Da in the fraction obtained upon stimulation. Thus, our data indicate that CRASP is a secretory protein whose release from the heart is regulated by neuronal inputs to the atrium.

### CRASP is not abundant in the haemolymph

To examine CRASP abundance in the haemolymph, haemolymph samples from 11 adult snails, weighing 46 g to 106 g, were analysed by LC-ESI MS. CRASP was detected as a 10-fold charged ion species with typical heterogeneity at *m/z* 1914.3, *m/z* 1896.3 and *m/z* 1831.9. A retention time of ~1660 s matched that observed for purified CRASP (Fig 9C). The concentration of CRASP in the haemolymph samples varied from 30 ng/ml (1.6 nM) to 400 ng/ml (20.8 nM) with a mean of 152 ± 121 ng/ml (8.0 ± 6.3 nM). No correlation with body weight was found. These results indicate that CRASP has only low abundance in the haemolymph.

## Discussion

Here, we have described the purification, cloning, characterization and expression pattern of CRASP, an 18.1-kDa protein secreted from the atria of the terrestrial pulmonate mollusc *A*. *achatina*. CRASP is an acidic, soluble 158-residue glycoprotein rich in cysteines, which form five disulphide bonds. We identified two CRASP isoforms, A and B, with CRASP-B differed by monoisotopic mass change of +0.97 Da and a pI shift of −0.07. CRASP-B most likely has a single amino acid substitution of Asn to Asp, because substitution of Gln to Glu shifts the pI by −0.05. Mass spectrometry also revealed that the CRASP heterogeneity most probably was due to the presence of varying numbers of hexose residues (+162 Da) linked to core glycan and varying numbers of methyl groups (+14 Da) linked to terminal hexoses, both well-documented features of N-glycosylation in gastropods [18]. Unfolding studies of CRASP monitored with ESI MS demonstrated that reductive cleavage of disulphide bonds is essential to make all basic residues available for protonation. The observed changes in the ESI charge state distribution following the reduction of disulphide bonds clearly indicated unfolding of the protein [19,20]. CD spectroscopy studies demonstrated that CRASP adopts a predominantly *α*-helical conformation with minor *β*-structure content. The amino acid sequence of CRASP did not show any significant similarity to known proteins.

### Taxonomically restricted distribution

The CRASP gene can be tentatively classified as an ‘orphan’ or taxonomically restricted gene because no similar sequences were found in closely related species [21,22]. However, our failure to detect similar sequences could be due to the fact that few mollusc species have a sequenced genome [23]. Indeed, only three gastropod species, *L*. *gigantea*, *A*. *californica* and *B*. *glabrata* have had their genomes sequenced; only transcriptome data are available for other species. Thus, at present, the extent of taxonomic restriction of CRASP remains an open question until genomic data from a more closely related species becomes available.

From a structural viewpoint, CRASP meets another criterion of taxonomically restricted genes, which often encode short proteins with a high amount of low complexity and disorder [24]. We detected a low-complexity region and short unstructured loops in the CRASP sequence using computational approaches, and CD spectroscopy revealed a relatively high content of disordered residues. Many taxonomically restricted genes are expected to be fast-evolving sequences [24]. In proteins encoded by taxonomically restricted genes, disulphide bonds stabilize tertiary structure, while the remainder of the sequence is free to diverge beyond recognition [25]. The disulphide bond pattern and cysteine spacing motif should be considered in future attempts to identify homologs of CRASP because these are conserved features in many protein families [26,27]. It has been suggested that taxonomically restricted genes play roles in the generation of taxon-specific features and morphologies [22]. We propose that CRASP plays a role in physiological processes specific for terrestrial pulmonates.

### Validity of the assigned fold

It has been proposed that the current PDB library is nearly complete, indicating that appropriate templates should be detectable for almost all single-domain proteins [28]. However, the available fold recognition approaches often fail to detect the correct template for targets with undetectable sequence similarity. The right fold should occur amongst the top 10 hits of a reasonable threading program [29]. Recently, it was shown that the addition of disulphide connectivity patterns as distance restraints improve the quality of QUARK *ab initio* structure prediction [30]. We combined 15 fold recognition methods, covering different categories of sequence to structure alignment algorithms, with *ab initio* folding simulation to show that CRASP likely shares structural similarity with members of the SCOP a.118.9 ENTH/VHS domain superfamily. This superfamily comprises compact domains of ~150 residues, formed by eight *α*-helices assembled in a right-handed superhelical fashion [31]. These domains share remarkable structural similarity despite little sequence conservation [32,33]. The superhelical fold of the ENTH/VHS domain superfamily is preserved by the conserved hydrophobic core [31]. The arrangement of helices creates a concave inner face formed by helices 2, 4 and 7, where helix 4 is the most deeply buried. The helices are linked by short turns, except for the loop between helices 7 and 8, which is the most flexible region [33]. Functional differences are produced by varying the solvent accessible residues involved in protein-protein interactions [32–34]. Our conclusion that CRASP folds into this structure is based on four lines of evidence: *(i)* CD spectroscopy study of secondary structure content, *(ii)* disulphide linkage, *(iii)* shape of the CRASP molecule and *(iv)* predicted buried residues and disorder.

- The CD data were analysed using four different deconvolution algorithms; all lead to similar solutions. The number and average length of the *α*-helical segments estimated from the CD spectra closely resembled those of the ENTH/VHS domains. Good agreement between the crystallographic data and spectroscopic estimates of the number and average length of the secondary structure segments was demonstrated [35,36]. The small content of *β*-structure predicted at the N-terminus should not significantly affect the overall right-handed superhelical topology.
- Cleavage of CRASP with trypsin produced several unique, single disulphide-bonded peptides from which we determined the disulphide bonding pattern. We searched for similar combinations of disulphide bonding patterns and cysteine spacing motifs among disulphide-linked all-alpha helical proteins. The barley lipid transfer protein (**PDB ID: 1BE2**) and human p8MTCP1 protein (**PDB ID: 1HP8**) both showed a similar combination of two enclosed bonds that constrain the right-handed superhelical packing of three helices and an alpha-hairpin, respectively (S5A and S5B Fig). Both super-secondary structural motifs fit well with the right-handed superhelical fold assigned to CRASP. The N-terminal disulphide bond Cys3-Cys8 creates a small loop in CRASP that may form a cap shielding the hydrophobic core, similar to the N-terminal leucine-rich repeat capping motifs (LRRNT) that are common in extracellular and membrane-associated leucine-rich repeat proteins [37]. The LRRNT cap notably contains an antiparallel *β*-sheet that might explain the small content of *β*-structure content in CRASP.
- Size-exclusion chromatography showed that the hydrodynamic radius of the CRASP molecule was higher than that expected for a globular protein of the same mass, indicating that CRASP is slightly elongated. This is in agreement with the assigned fold because most structures with a right-handed superhelical fold are elongated to some extent [38].
- There was a good match between CRASP buried residues and the residues involved in hydrophobic core packing in the templates we identified. This is most evident from the match between the fourth helix of the ENTH/VHS domains and the 19-residue predicted buried region of CRASP. Similarly, the predicted disordered loop in CRASP matched the long loop between the seventh and eights helices of the Pcf11 domain (**PDB ID: 1SZ9**), reported to be the most flexible region [33].

Despite the correspondence between the modelling results and biophysical measurements, the possibility that CRASP has another, perhaps novel, fold cannot be ruled out. Only determination of the 3D structure will confirm or reject our assignment.

### Putative physiological role of CRASP

We found a tight association between CRASP expression and the circulatory system (atrium, vein and aorta). Not all heart tissues displayed high levels of expression: the low expression in the ventricle myocardium indicates that the tissue specificity of CRASP is related to its function. In situ hybridization showed intense labelling in large globular cells in the atrium, which were clearly identified as atrial granular cells based on morphological features [11,14]. The rarely observed cells with a high nuclear-cytoplasmic ratio could be granular cell progenitors. The granular cells in the *Achatina* atrium are ideally placed for secretion; therefore, once released, CRASP could easily spread through entire circulatory system and quickly reach peripheral targets. Indeed, the circulation time of the haemolymph in pulmonates ranges from 4–6 min [2]. Surprisingly, we found that substantial amounts of CRASP were secreted by the atrium into the haemolymph following nerve stimulation. Furthermore, CRASP was the most, if not the only, abundant protein among those released. Indeed, the quantity of CRASP released was ~40 μg per heart, representing ~1% of the atrium dry weight. Assuming a shell weight of 17.5 ± 1.2% of the total body weight and a blood volume of 40.3% of the wet weight without shell [39], we estimate that CRASP could reach a maximum level of 50–100 nM (1–2 μg/ml) in the haemolymph after total degranulation of atrial granular cells. The level of CRASP measured in intact snails was in the range of 1.6–20.8 nM, which is comparable to the effective concentration of *Lymnaea* epidermal growth factor that can induce neurite outgrowth from snail neurons *in vitro* at 22 nM [40]. However, the effective concentration of *Lymnaea* cysteine-rich neurotrophic factor is reported to be much lower (25 pM) [41], and picomolar concentrations of *Lymnaea* large cardioactive peptide have been reported in the haemolymph [42]. Given that the CRASP level observed in intact snails was higher than that expected for growth factors and neurohormones, it seems unlikely that they have similar functions. The level of C-reactive protein abundant in the haemolymph of *A*. *fulica* ranges from 1–5 mg/ml [43], while total haemolymph protein in *Achatina* ranges from 1.5–16.3 mg/ml [39]. Our data indicate that while CRASP is not abundant in the haemolymph, its high abundance in the atrium is sufficient to increase its level in the haemolymph significantly. Our data further indicate that the secretory activity of granular cells is regulated by neuronal inputs. Both its high abundance in the atrium and the regulation of its release by the nervous system suggest an important role for CRASP in *Achatina* physiology. Moreover, the expression of CRASP in newborn and adult snails suggests its involvement in a basic physiological process.

The high expression observed in the pericardium and nephridium suggests the involvement of CRASP in processes related to excretion. Indeed, in gastropods, the atrium, pericardium and nephridium all play excretory roles [44]. It is tempting to conclude that CRASP has a role in chemical communication, similar to mammalian major urinary proteins (MUPs) which are expressed at the sites of pheromone excretion (liver and kidney). MUPs bind volatile hydrophobic pheromone ligands and release them from urine-derived scent marks [45,46]. Gastropods rely on olfaction and chemoreception in the perception of distance and near objects. Chemoreception in gastropods controls not only mating, but also homing and aggregation [47].

Structural features also suggest possible functions for CRASP. Small disulphide-rich proteins are predominantly extracellular, and their related set of functions includes signalling (hormones, growth factors) and binding (enzyme inhibitors, toxins, defensins) [48]. A similar set of functions was predicted for CRASP using a homology-independent protein feature-based method, FFPred: receptor binding, enzyme binding, transport, cytokine activity, growth factor activity, enzyme regulator activity and cell surface receptor signalling pathway. We performed a set of pilot experiments to test the possible functions of CRASP: antibiotic activity against *Escherichia coli* and *Pseudomonas aeruginosa* by the disc diffusion susceptibility method; opsonization of heat-inactivated, FITC-labelled yeasts and phagocytosis by snail haemocytes; agglutination and haemolysis of human erythrocytes; and neurotrophic effect on a primary culture of snail neurons. Unfortunately, all experiments yielded negative results.

The probable structural similarity to the ENTH/VHS domain superfamily supports a protein-binding function for CRASP because protein binding is a general function of these domains. Furthermore, an all-alpha, right-handed superhelical fold is representative of proteins with solenoid topology, most of which are known to be involved in protein-protein interactions [49]. Haemolymph proteins with all-alpha, right-handed superhelical fold that bind small hydrophobic ligands are known in insects: the N-terminal domain of *Bombyx mori* lipoproteins [50,51] and insect chemosensory proteins [52]. Based on the available data, we propose that CRASP adopts an all-alpha, right-handed superhelical fold and plays a ligand-binding role in the snail haemolymph. The nature of the ligand, as a protein or small hydrophobic molecule, and the biological activity of CRASP remain to be elucidated.

In conclusion, this study showed that the molluscan atrium expresses and releases a specific protein into the circulation, suggesting that the physiological roles of the molluscan heart are more diverse than previously thought and not yet clearly understood. We propose that CRASP is a representative member of a taxonomically restricted protein family specific to terrestrial pulmonates that plays an important role in snail physiology. Because of its novelty and structural features, CRASP is an interesting subject for further structural and functional characterization.

## Materials and Methods

### Ethics statement

All experiments were conducted in accordance with the national legislation (Article 245 of the Criminal Code of Russian Federation "Animal abuse"). Steps were taken to ensure that animals did not suffer unnecessarily at any stage of the experiments. Tissue samples were excised quickly and the animals were euthanized by rapid freezing. Some animals were used only for the collection of haemolymph samples, and their shells were sealed with wax after the procedure.

### Animals

Experiments were carried out on the adult giant Ghana snails, *Achatina achatina* Linnaeus (1758; Gastropoda) bred in the laboratory. The animals were kept in a terrarium at 25°C, on a 12h:12h light:dark cycle, and were fed a vegetable diet supplemented with oat flakes and chalk.

### Purification of CRASP from atria

The atria were excised, lysed in 0.5% Triton X-100, 5 mM EDTA, 100 mM NaCl, 50 mM Tris/HCl, pH 7.5 with a protease inhibitor cocktail (#P2714, Sigma) and clarified by centrifugation at 14000 g for 10 min. The 0.5 ml of supernatant was loaded onto a Superdex 200 10/300 GL size exclusion column (GE Healthcare) equilibrated in 50 mM NaCl, 20 mM Tris/HCl, pH 7.5. The fraction from 31 to 34 min at a flow rate of 0.5 ml·min^-1^ was loaded onto a Mono-Q HR 5/5 anion exchange column (Pharmacia Fine Chemicals) equilibrated with the same buffer, and the bound proteins were eluted with a linear gradient of 250 mM NaCl, 20 mM Tris/HCl, pH 7.5 for 10 min at a flow rate of 1 ml·min^-1^.

Two isoforms of CRASP were isolated after anion exchange chromatography. These native isoforms were used for subsequent analysis by isoelectric focusing, analytical size exclusion and CD spectroscopy. To further purify the isoforms, two major peaks were collected and applied to a ProRPC HR 5/2 C4 reversed phase column (Pharmacia Fine Chemicals). The proteins were eluted with a linear gradient of 50% n-propanol, 0.1% trifluoroacetic acid (TFA) in water for 20 min at 0.5 ml·min^-1^. Purified CRASP isoforms were collected and lyophilized. In all cases, the CRASP concentrations were determined using A_280 nm, 1 mg·ml_^-1^ of 1.243 litre g^-1^ cm^-1^ (assuming all cysteines are oxidized) calculated with a ProtParam on the ExPASy server [53]. SDS-PAGE and silver staining were used to visualize all purification steps. Protein purity and identity was confirmed by LC-ESI MS.

### SDS-PAGE

SDS-PAGE was performed on 15% gels with a Mini-Protean II electrophoretic cell (Bio-Rad). Unstained Protein MW marker (Thermo Scientific) was used. To visualize proteins, the gel slabs were silver-stained [54].

### Protein sequencing

For N-terminal sequencing, protein bands from SDS-PAGE were transferred to Immobilon-PSQ membrane in 50 mM borate buffer pH 9.0 containing 10% methanol (v/v) at 25 V for 2 h. Then, the membrane was stained with Coomassie Brilliant Blue G 250 to localize the protein bands. For internal sequencing, cystines were reduced with dithiothreitol, alkylated with iodoacetamide and the protein was digested with ArgC endoproteinase. HPLC purified peptides and excised protein bands were sequenced via automated Edman degradation using a Procise Model 492 Edman Micro sequencer (Applied Biosystems) at the Division of Clinical Biochemistry of Medical University of Innsbruck, Austria.

### CRASP cDNA cloning and sequencing

Total RNA was extracted from the atria of *A*. *achatina* using TRI Reagent (Sigma) and reverse-transcribed using a MINT cDNA synthesis kit (Evrogen) in accordance with the manufacturer’s instructions. CRASP cDNA was identified by 3′ and 5′ rapid amplification of cDNA ends using the MINT RACE cDNA Amplification Set (Evrogen) as described in the manufacturer’s instruction. For 3′ RACE, nested degenerate oligonucleotide primers were designed on the basis of the determined amino acid sequences of the N-terminal and internal peptide fragments, using the iCODEHOP algorithm [55]. Sequences of the primers are listed in S1 Table. Three rounds of touchdown amplification were carried out with the Nterm_F, Int1_F and Int2_F primers. The annealing temperature was decreased by 1°C every second cycle from 65°C to 55°C. The amplified product was cloned in a pCR II vector, using the Dual Promoter TA Cloning Kit (Invitrogen), and sequenced with a 3130 Genetic Analyzer using Big Dye Terminator sequencing reagent (Applied Biosystems). For the 5′ RACE reaction a pair of nested gene-specific primers was designed from the sequence of the 3′ RACE product (NCS1_R, NCS3_R) and the amplification product was cloned and sequenced. Finally, a set of gene-specific primers was used (NCS1_F, NCS1_R) and the full-length CRASP cDNA was amplified with High Fidelity PCR enzyme Mix (Thermo Scientific) and cloned. Five clones were selected and sequenced in both directions.

### Analytical isoelectric focusing

Analytical isoelectric focusing was carried out under native conditions on 5% polyacrylamide gel slabs that were 0.8 mm thick. The gels contained 10% w/v sucrose and 4% v/v mixture of Ampholyte 3–6 and Pharmalyte 3–10 (1.6:1 v/v). Cathode and anode solutions were 0.5 M NaOH and 0.5 M H_3_PO_4_ respectively. The electrode strips were placed on the gel 8 cm apart. The apparatus was cooled with circulating water at 4°C. After prefocusing at 1200 V for 1400 Vh to develop the pH gradient, samples were loaded at 500 V for 30 min and separated at 1600 V for 3600 Vh. Gels were stained with Coomassie Brilliant Blue G 250. A calibration equation was obtained from an sigmoidal fit of relative mobility as a function of the isoelectric point (pI), using the Low range Amersham calibration kit (GE Healthcare) of six proteins with known pI values, between 3.50 (amyloglucosidase) and 6.55 (human carbonic anhydrase B), and this was used to estimate the pI value of CRASP.

### Deglycosylation of purified CRASP

Twenty μg of each of the isoforms purified by RP-HPLC were dissolved in 20 mM ammonium bicarbonate. A 30 μg sample of the native isoform A was rechromatographed on Superdex 200 10/300 GL in PBS. Then, each sample was incubated with 15 units of peptide N-glycosidase F (F8435, Sigma) in a total volume of 200 μl at 37°C for 18 h. The deglycosylated protein samples were analysed using LC-ESI MS and size exclusion chromatography.

### Size exclusion chromatography

A Superdex 200 10/300 GL size exclusion column (GE Healthcare) was equilibrated at 20°C in 20 mM phosphate buffer, pH 7.5, containing 280 mM NaCl and 0.02% sodium azide, at a flow rate of 0.5 ml·min^-1^. The void volume (V_0_ = 8.09 ml) and total volume (V_t_ = 21.22 ml) of the column were determined by injection of blue dextran and acetone, respectively (sample volume 200 μl). After this the elution volumes (V_e_) were measured for standard proteins with known hydrodynamic properties and for natively deglycosylated CRASP. Hydrodynamic properties and Stokes radii (R_s_) of standard proteins were taken from references [56–65] and are listed in S2 Table. The partial specific volume (ῡ) was calculated with Sednterp (http://sednterp.unh.edu/), a ῡ of 0.719 ml·g^-1^ was considered for CRASP. The standard proteins were catalase (V_e_ = 13.31 ml), aldolase (V_e_ = 13.62 ml), bovine serum albumin (V_e_ = 14.59 ml), ovalbumin (V_e_ = 15.61 ml), carbonic anhydrase (V_e_ = 16.70), chymotrypsinogen A (V_e_ = 17.34 ml), ribonuclease A (V_e_ = 17.88 ml) and bovine pancreatic trypsin inhibitor (V_e_ = 19.12 ml). For each protein, a distribution coefficient (K_D_) was calculated. A calibration equation was obtained from an exponential decay fit of the K_D_ as a function of the R_s_ and used to estimate the R_s_ of CRASP. The frictional ratio was calculated by using the deduced R_s_ and the minimal radius of a sphere that contains a given mass of protein [66].

### Protein extraction from the hemolymph

One-milliliter samples of haemolymph were collected from the pulmonary vein of 11 untreated adult snails, weighing from 46 g to 106 g. One ml of anticoagulant buffer (61 mM NaCL, 3.3 mM KCl, 40 mM EDTA, 10 mM Hepes, pH 7.5) supplemented with 100 μM PMSF was added to each sample, and the samples were centrifuged at 3000 rpm for 5 min in a MiniSpin plus centrifuge (Eppendorf). Then, 20 μl of glacial acetic acid was added to each sample and 2-ml samples were applied to the Strata C18-T 100 mg solid-phase extraction tubes (Phenomenex) equilibrated with 10% v/v acetonitrile and 0.1% v/v TFA. Unbound proteins were removed by washing with 5 ml of equilibration buffer, and bound proteins were eluted with 1.5 mL of 90% v/v acetonitrile and 0.1% v/v TFA. The samples were dried with a rotor vacuum evaporator, redissolved in 100 μl of aqueous 0.1% v/v acetic acid containing 0.1 nM of the bombesin mass standard, and analysed with LC-ESI MS.

### LC-ESI MS analysis of CRASP

A microbore HPLC system (MiLiChrom A-02, EcoNova) coupled in-line with an ESI-oa-TOF mass spectrometer (MCH-5310, Institute of Analytical Instrumentation RAS) was used for the LC-ESI MS analyses. Proteins were separated with a Jupiter C5 reversed phase column (2 mm × 100 mm, 5 μm, 300 Å, Phenomenex) using mobile phases A (0.25% v/v formic acid in water) and B (0.25% v/v formic acid in acetonitrile). A linear gradient of 35–55% B over 38 min was followed by 55–90% B for 4 min at a flow rate of 100 μl·min^-1^. The ESI source was operated in positive ion mode with a desolvation gas temperature of 280°C, a capillary voltage of 3.2 kV, and a cone voltage of 120 V. Monoisotopic peaks of singly and doubly charged bombesin forms with *m/z* 810.415 a.m.u. and *m/z* 1619.82 a.m.u. were used for mass calibration. Data were analysed using TOF Explorer 2.1 software (Institute of Analytical Instrumentation RAS). The CRASP was identified at the retention time observed for purified protein by monitoring its 10 charged forms. For quantitative analysis, the intensity of the most abundant charged form with *m/z* 1914.4 was integrated by retention time from 1600 ± 50 s to 1750 ± 50 s and by a *m/z* range of 1912–1917 a.m.u. The background noise was estimated as a mean of intensities in the m/z ranges of 1855–1860 a.m.u. and 1970–1975 a.m.u. A calibration equation was obtained from an exponential growth fit of the integrated intensity as a function of the CRASP amount in five calibration samples, ranging from 15 ng to 300 ng, and used to estimate the CRASP amount in the haemolymph samples. The data are presented as mean ± SD. The calibration plot is shown in S6B Fig.

### Determination of the total number of disulphide-bonded and free sulphhydryl groups

Deglycosylated CRASP (50 μg) was denatured in 100 μl of 6 M guanidinium chloride, 50 mM NaH_2_PO_4_, pH 6.5 for 30 min at 37°C. Then, the protein was subjected to reduction with 0.26 μM dithiothreitol for 1 h at 37°C followed by alkylation with 2.9 μM iodoacetamide for 1 h at 37°C. As a control, after denaturation, the protein was alkylated without reduction. The samples of native, reduced, reduced/alkylated and non-reduced/alkylated protein were run on a LC-ESI MS instrument as described above. Carboxamidomethylation of a cysteine residue increase an average mass by 57.05 Da.

### Determination of the disulphide-bonding pattern

Samples of deglycosylated CRASP isoforms were run on SDS-PAGE gels without reduction and stained with Coomassie Brilliant Blue G 250. Bands of interest were excised from the gel and subjected to digestion with Proteomics Grade Trypsin (Sigma). Peptides were extracted from the gel matrix and subjected to LC-ESI MS analysis. Tryptic digests were separated with a Jupiter Proteo C12 reversed phase column (4 μm, 90 Å, 1 mm × 50 mm, Phenomenex) using mobile phases A (0.25% v/v formic acid in water) and B (0.25% v/v formic acid in acetonitrile). A linear gradient of 20–50% B over 40 min was followed by 50–90% B for 10 min at a flow rate of 50 μl·min^-1^. The ESI source was operated in positive ion mode with a desolvation gas temperature of 280°C, capillary voltage of 3.2 kV, and cone voltage of 100 V. To confirm the disulfide pattern, tryptic digests were reduced with dithiothreitol and immediately subjected to LC-ESI MS as described above.

### CD spectroscopy

Far UV CD spectra were recorded from 190 nm to 260 nm on a Jasco J-810 spectropolarimeter at 0.2 nm intervals, using a 0.1 cm optical pathlength cuvette. For optimal spectra, CRASP isoforms were rechromatographed on a Superdex 200 10/300 GL size exclusion column in 100 mM NaF, 5 mM NaH_2_PO_4_, pH 7.1. Protein concentrations were determined using a Hitachi U-3900H UV-Visible spectrophotometer by absorption at 280 nm. The molar extinction coefficient of 22515 M^-1^ cm^-1^ was calculated using ProtParam. CD data were deconvoluted with K2D3 webserver [67] and with algorithms SELCON3 [68], CDSSTR [69], and CONTIN/LL [70] implemented in a DICHROWEB webserver [71]. Protein reference data set SP175 [72] was used for the DICHROWEB calculations. The number and average length of *α*-helical and *β*-strand segments were estimated according to Sreerama et al. [35]. A normalized standard deviation (NRMSD) was calculated as described by Mao et al. [73].

### Sequence analysis and database searches

Similarity searches in UniProtKB and the NCBI non-redundant sequence databases were performed using BLASTP [74], CS-BLAST [75], HMMER [76] and HHblits [77] under default settings. TBLASTN searches were performed against molluscan genomes and mollusc-restricted transcriptome databases available at the NCBI server. Gene Ontology terms were predicted with the homology-independent protein feature-based method FFPred [78]. The potential N-glycosylation site was predicted with NetNGlyc [79]. The low compositional complexity region was identified with SEG [80]. Secondary structure was predicted with PSIPRED 3.3 [81], Porter 4.0 [82], Jpred 4 [83] and the GeneSilico metaserver (https://genesilico.pl/meta2). Relative solvent accessibility was predicted with SPINE-X [84]. Short disordered regions were predicted with POODLE-S [85] and VSL2B [86]. All secondary structure predictions were made after removal of the signal peptide. The average molecular mass and isoelectric point of CRASP were calculated with Compute pI/MW, ans the monoisotopic masses of the tryptic peptides were calculated with PeptideMass on the ExPASy server [53].

### QUARK-based fold recognition

An approach suggested and validated by Zhang’s group [16,17] was used. Briefly, since threading and *ab initio* folding simulations are two distinct approaches, any reasonable match of the experimental structure identified by threading with a model (TM-score > 0.35) may indicate a correct template hit. Thus, for low homology targets, *ab initio* model can be used to identify the correct fold from the pool of templates identified by threading programs. Threading was conducted by the following fold recognition programs under default settings: SPARKS-X [87], FUGUE [88], pGenTHREADER [89], RaptorX [90], IntFOLD2 [91], PROCAIN [92], Phyre2 [93] and the LOMETS meta-threading server [94], which included MUSTER [95], SP3 [96], PROSPECT II [97], SAM-T02 [98], HHSerach [99], FFAS [100], PRC [101] and PPA-I [102]. The same template structure identified with different programs was processed as a different hit. The QUARK [103] server was used for *ab initio* folding directed by spatial restraints derived from the experimentally determined disulphide bonding pattern. A Cα-Cα′ distance of 5.6 Å was considered [104]. Then, we compared the top QUARK model refined with ModRefiner [105] to the top 10 templates identified by each threading programs and then re-ranked all templates based on their TM-score and p-value calculated with TM-align [106] and FATCAT in rigid mode [107] correspondingly. Models were displayed using UCSF CHIMERA [108].

### Quantitative real time PCR

To determine tissue expression patterns of CRASP, total RNA was extracted from the following tissues and organs: the atrium, ventricle, pericardium, pulmonary vein, lung plexus, arteria anterior, nephridium, intestine, digestive gland, connective tissue, columellar retractor, cerebral ganglion, subesophageal ganglion, pedal nerves, ovotestis, diaphragm (floor of mantle cavity) and the hemocyte pellet, using a GeneJET RNA purification kit (Thermo Scientific). RNA was sampled from two individual snails, weigh 62 g and 73 g, and processed together as a single data point for each tissue and organ. RNA was also extracted from one newborn snail weighing 95 mg. RNA quantities were measured with a NanoDrop 1000 spectrophotometer (Thermo Scientific). Only RNA samples with an A_260_/A_280_ ratio >1.8 and A_260_/A_230_ ratio >2.0 were used for analysis.

The first-strand synthesis was carried out with a RevertAid First Strand cDNA Synthesis Kit (Thermo Scientific) using 1 μg of DNase I- (Thermo Scientific) treated total RNA as the template. Each cDNA sample was diluted 1:16 and used as a template for qRT-PCR analysis with the Luminaris Color HiGreen Low ROX qPCR Master Mix kit (Thermo Scientific) using the manufacturer’s protocol.

The alpha-tubulin and 60S acidic ribosomal protein P0 (60SARP) genes were used to normalize relative gene expression. Orthologues of *Limax valentianus* alpha-tubulin (AB099707.2) and *Haliotis diversicolor* 60SARP (EU244370.1) genes were identified by tBLASTn searches of the EST database restricted to the *Sigmurethra* taxonomic category of land snails. The alpha-tubulin primers were designed based on a consensus of cDNA sequences from *Mandarina ponderosa* (DR044666.1), *Nesiohelix samarangae* (DC604631.1) and *Limax valentianus*. The 60SARP primers were designed based on *M*. *ponderosa* (DR044714.) and *N*. *samarangae* (DC604437.1) cDNA sequences. The primer pairs used are listed in S1 Table.

The analysis was performed on an Applied Biosystems 7500 real-time PCR System (Applied Biosystems). Thermal cycling conditions were: initial uracil-DNA glycosylase pretratment for 2 min at 50°C and polymerase activation for 10 min at 95°C followed by 40 cycles of 95°C for 15 s, 60°C for 30 s, 72°C for 40 s. Duplicate reactions per cDNA sample were performed. Melting curve analysis and agarose gel electrophoresis was performed to confirm the specificity of the amplification reactions. The raw amplification data (i.e., not baseline-corrected) were analyzed using LinRegPCR [109] as described by Ruijter et al. [110]. The resulting starting concentrations of CRASP amplicon were obtained and normalized to that of the reference genes. The data were presented as fold change of CRASP gene expression level relative to a sample with minimal expression level.

### Whole mount RNA FISH

The heart was exposed and prefixed *in situ* by injection of 4% PFA in PBS, adjusted to 200 mOsm, into a pulmonary vein. The atrium was then excised, fixed with the same fixative for 2 hr at 4°C and washed in PBS. Atria were pretreated with 2 μg·ml^-1^ proteinase K (Thermo Scientific), 0.1% SDS in PBS for 4 min at 25°C and then the proteinase K was inactivated by incubation with 200 μM PMSF in 2×PBS for 1 min. The atria were postfixed in 4% PFA for 30 min, washed in 4×SSC and prehybridized in 1% dextran sulfate, 50% formamide, 100 μg·ml^-1^ *E*. *coli* tRNA (Roche), and 4×SSC for 1 hr at 35°C. A synthetic 25-mer 5’-end biotin-labeled oligonucleotide probe, representing a part of the coding region of the CRASP gene, was used (5′ TTTGACAGTCCAAGCACTCCTACAC-3′). Hybridization was performed with 0.5 μM of probe for 18 hr at 35°C. Samples were washed four times at 35°C in 50% formamide, in 4×SSC for 15 min and four times at 45°C in 0.2×SSC, 0.1% Tween 20 for 15 min. After blocking in 3×PBS containing 1% BSA and 0.1% Tween 20, the probe was detected using avidin-Cy3 (1:400, Jackson Immuno Research) and biotinylated goat anti-avidin antibody (1:400, Vector Laboratories) followed by avidin-Cy3 (1:400). Avidin and antibody were diluted in 3×PBS containing 1% BSA, 0.1% Tween 20. After each incubation step the samples were thoroughly washed in PBS-Tween. Samples were counterstained with DAPI and mounted in VectaShield (VectorLaboratories). Fluorescent images were taken on a Zeiss Axioscope fluorescence microscope equipped with a Leica DFC 420 CCD camera. Confocal laser microscopy was done using a LEICA TCS SP5 CLS microscope. Negative controls included hybridization with RNase-treated atria (100 μg·ml^-1^ for 1.5 hr at 60°C after proteinase K treatment) and hybridization without probe.

### Stimulation of secretion and heart perfusion

All experiments were performed at room temperature. Six adult animals weighing from 56 g to 116 g were used. The shell was removed and the cardiorenal complex was surgically isolated with the pericardial branch of the intestinal nerve (heart nerve) length of 10–15 mm and fixed in the dissecting chamber with pins. The first cannula was inserted into the main pallial vein and tied at the entrance to the atrium and the second one at the ventricular apex. The heart nerve was partially desheathed and hooked to bipolar electrodes connected to an electrical stimulator (Accupulser Signal Generator, WPI).

After the preparation, the dissecting chamber was filled with physiological solution (61 mM NaCl, 3.3 mM KCl, 10.7 mM CaCl_2_, 13 mM MgSO_4_, 10 mM Hepes, pH 7.4) and the electrodes were raised above the surface by about 5 mm. The heart was perfused with physiological solution, using a Harvard Apparatus Model 11 syringe pump (Harvard Apparatus) with a constant flow rate of 0.3 ml·min^-1^. Three consecutive 1-ml fractions of perfusion fluid were collected before the stimulation. During square-wave electrical stimulation (5 V, 2 ms, 10 Hz) of the heart nerve, two consecutive 1 ml fractions of perfusion fluid were collected. All fractions were collected in 1.5 ml microtubes, containing 40 μl of 0.5 M EDTA pH 7.5, and placed in pre-chilled IsoFreeze rack (GeneMate).

Six independent experiments were performed. Fractions were centrifuged at 3000 rpm for 5 min with a MiniSpin plus centrifuge (Eppendorf) and 100 μl of each was analysed on a microbore HPLC system (MiLiChrom A-02, EcoNova). Proteins were separated with a Jupiter C5 reversed phase column (2 mm × 100 mm, 5 μm, 300 Å, Phenomenex) using mobile phases A (0.1% v/v TFA in water) and B (0.1% v/v TFA in acetonitrile). A linear gradient of 25–55% B over 17.5 min was followed by 55–90% B for 3 min at a flow rate of 200 μl·min^-1^. Absorbance was monitored at 216 nm, 250 nm and 280 nm. CRASP was identified at the retention time observed for purified protein and by separate LC-ESI MS analysis of selected samples. Data were analyzed with MultiChrom 1.5 software (Ampersand). A calibration equation was obtained from a linear fit of the peak area as a function of the CRASP amount in the eight calibration samples, ranging from 0.015 μg to 7.5 μg, and used to estimate the amount in the fractions of perfusate. A calibration plot is shown in S6A Fig. After stimulation, the atria and ventricles were isolated, blotted with filter paper and weighed. Then, they were dried at 60°C for 18 h and weighed again. The data are presented as mean ± SD.

## Supporting Information

S1 Fig Representative size-exclusion chromatograms supporting preservation of the compact native state of CRASP. First size-exclusion purification step (black); fractions of CRASP-A (red) and CRASP-B (green) pooled from the anion exchange purification step; rechromatographed sample of deglycosylated protein (blue). Note that removal of glycan increased the retention time by reducing the hydrodynamic radius of the protein.
(TIF)

S2 Fig Melting curve analysis. Amplification plots (top) and melting curves (down) of CRASP (brown), alpha-tubulin (blue) and 60SARP (green) for 18 samples analysed.
(TIF)

S3 Fig Agarose gel electrophoresis showing amplification of unique products for each gene in different tissues. The relative mobilities matched to those expected for amplicons of 120 bp, 226 bp and 345 bp for alpha-tubulin, CRASP and 60SARP respectively. Left gel: the atrium, ventricle and pericardium. Right gel: the intestine, connective tissue and columellar retractor.
(TIF)

S4 Fig qRT-PCR quantification cycle values of genes analysed in different tissues. The distribution is shown in a vertical box plot as median (lines), 25th to 75th percentiles (boxes) and range (whiskers) for 18 samples analysed.
(TIF)

S5 Fig Super-secondary structural motifs constrained by disulphide bonding patterns similar to those observed in CRASP. (A) Fragment of barley lipid transfer protein with a right-handed superhelical motif constrained by the enclosed disulphide bonds Cys3-Cys50/Cys13-Cys27. Note the asymmetrical cysteine spacing motif Cys-9-Cys-13-Cys-22-Cys. (B) Cysteine alpha-hairpin motif of the human p8MTCP1 protein, stapled with the enclosed disulphide bonds Cys7-Cys38/Cys17-Cys28. Note the symmetrical cysteine spacing motif Cys-9-Cys-10-Cys-9-Cys.
(TIF)

S6 Fig Calibration plots used for quantitative analysis of CRASP. (A) Calibration plot used to estimate the amount of CRASP in fractions of perfusion fluid with HPLC. The equation was obtained from a linear fit of the peak area as a function of the CRASP amount in eight calibration samples, ranging from 0.015 μg to 7.5 μg. (B) Calibration plot used to estimate the amount of CRAS in haemolymph samples with LC-ESI MS. The equation was obtained from an exponential growth fit of the integrated intensity as a function of the CRASP amount in five calibration samples, ranging from 15 ng to 300 ng. The calibration samples were analysed before (filled circles) and after (open circles) measurement of the experimental samples.
(TIF)

S1 Table Oligonucleotide primers used in the study. TD touch down.
(DOC)

S2 Table List of structural parameters of standard proteins considered in this study. The Stokes radius (*R*_*S*_) was calculated from the experimental translational diffusion coefficient (*D*_t (20, W)_) or sedimentation coefficient (*S*_20, W_). Protein Data Bank files used for the calculation of molecular mass and partial specific volume (*ῡ*_20_) are shown. (*f/f*_min_) frictional ratio. References: a—[60], b—[65], c—[58], d—[62], e—[56], f—[63], g—[59], h—[57], i—[64], j—[61].
(DOC)
