# Supplementary figures and images for: Cysteine-Rich Atrial Secretory Protein from the Snail Achatina achatina: Purification and Structural Characterization
Source: PLoS One. 2015 Oct 7;10(10):e0138787. doi: 10.1371/journal.pone.0138787 (PMC4596865; doi:10.1371/journal.pone.0138787)

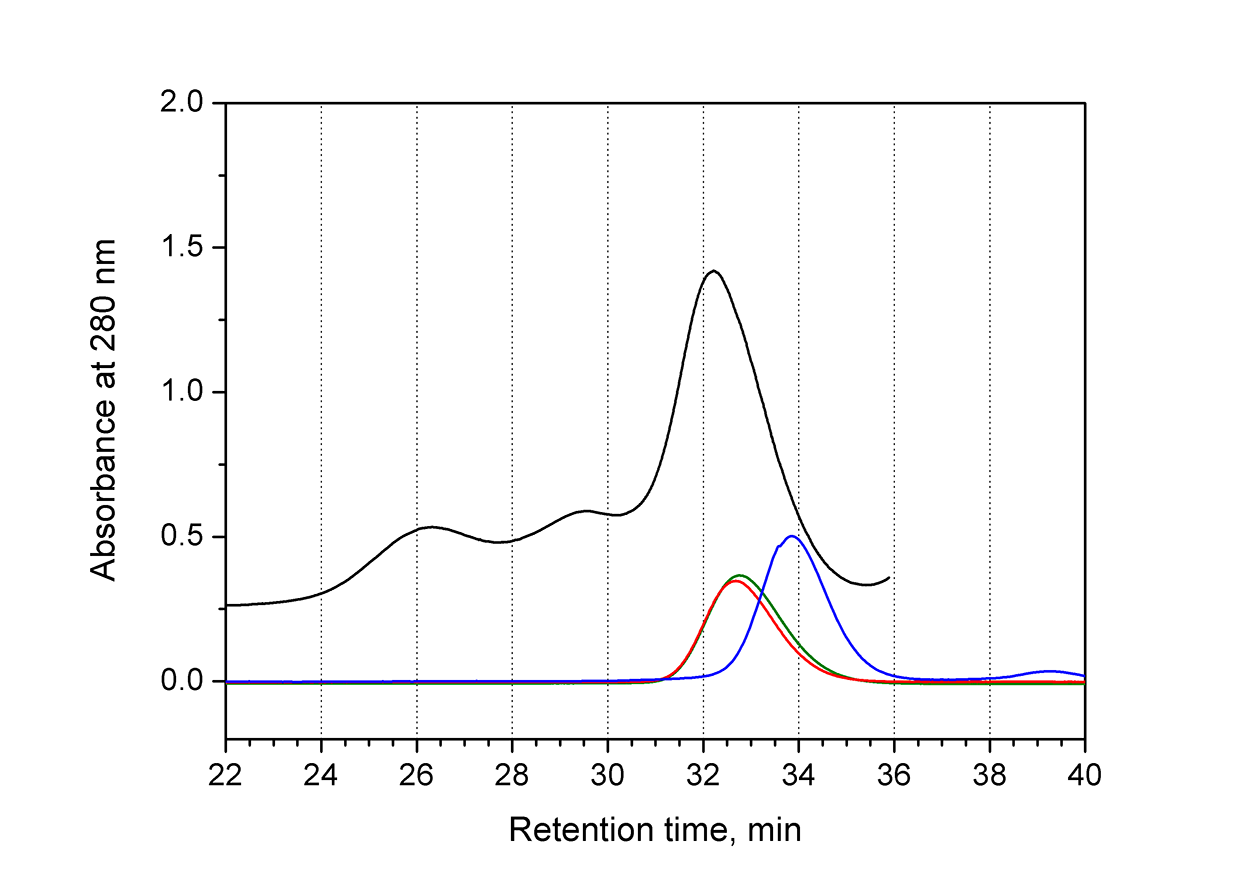

Supplement: S1 Fig — First size-exclusion purification step (black); fractions of CRASP-A (red) and CRASP-B (green) pooled from the anion exchange purification step; rechromatographed sample of deglycosylated protein (blue). Note that removal of glycan increased the retention time by reducing the hydrodynamic radius of the protein. (TIF) [file pone.0138787.s001.tif]

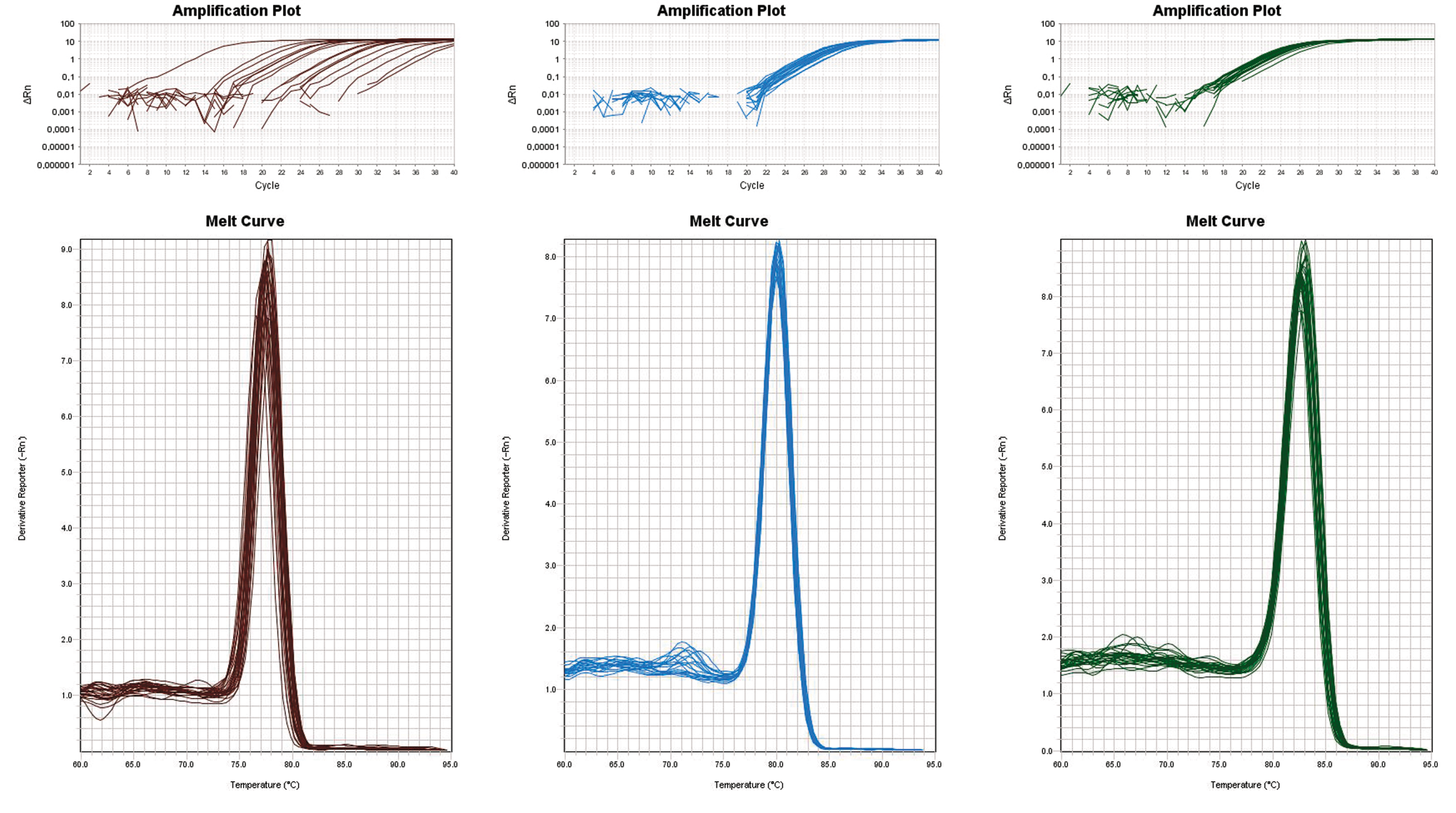

Supplement: S2 Fig — Amplification plots (top) and melting curves (down) of CRASP (brown), alpha-tubulin (blue) and 60SARP (green) for 18 samples analysed. (TIF) [file pone.0138787.s002.tif]

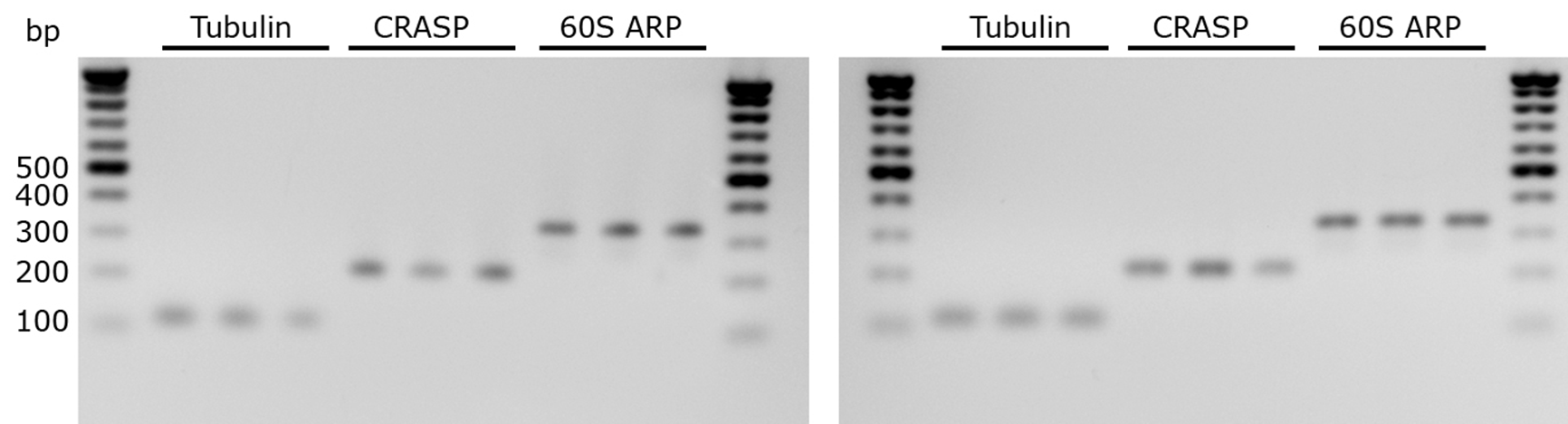

Supplement: S3 Fig — The relative mobilities matched to those expected for amplicons of 120 bp, 226 bp and 345 bp for alpha-tubulin, CRASP and 60SARP respectively. Left gel: the atrium, ventricle and pericardium. Right gel: the intestine, connective tissue and columellar retractor. (TIF) [file pone.0138787.s003.tif]

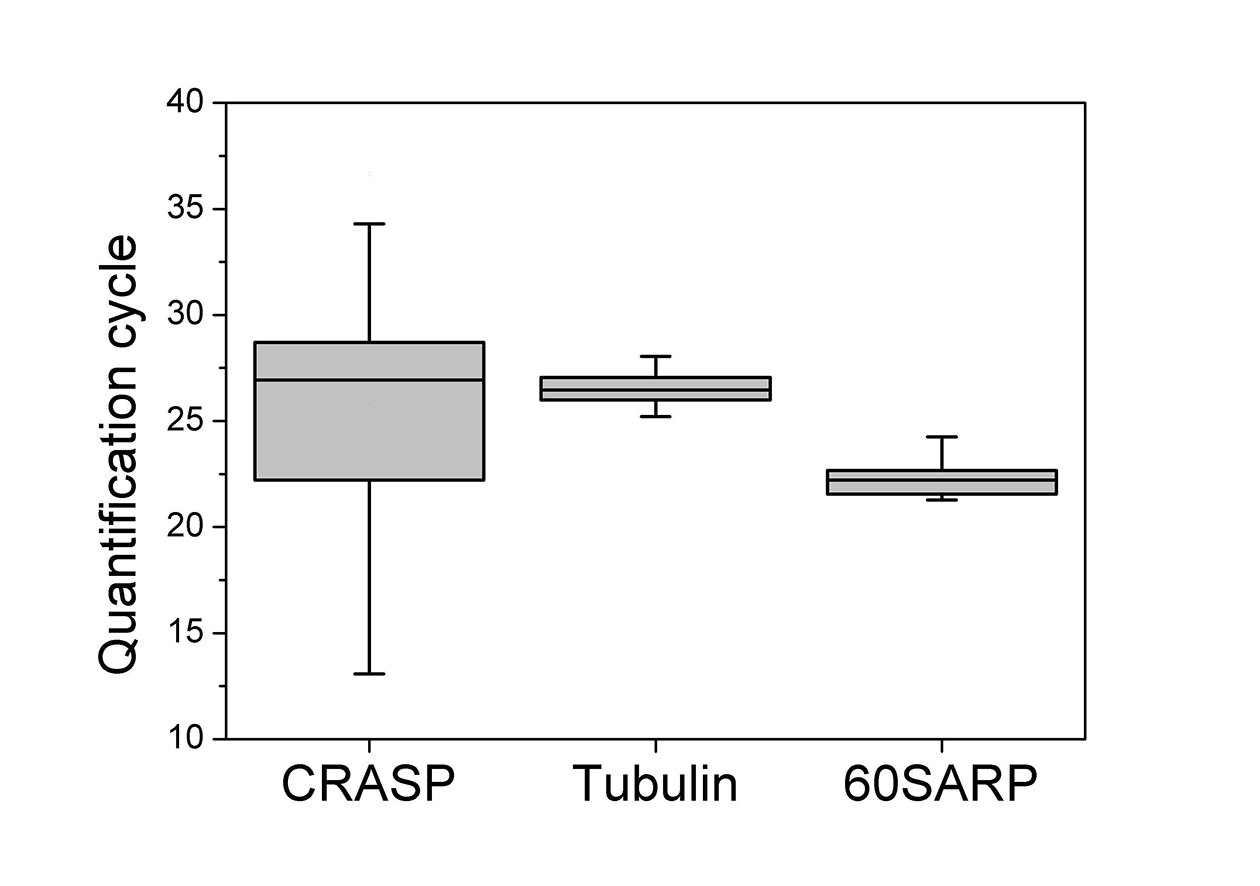

Supplement: S4 Fig — The distribution is shown in a vertical box plot as median (lines), 25th to 75th percentiles (boxes) and range (whiskers) for 18 samples analysed. (TIF) [file pone.0138787.s004.tif]

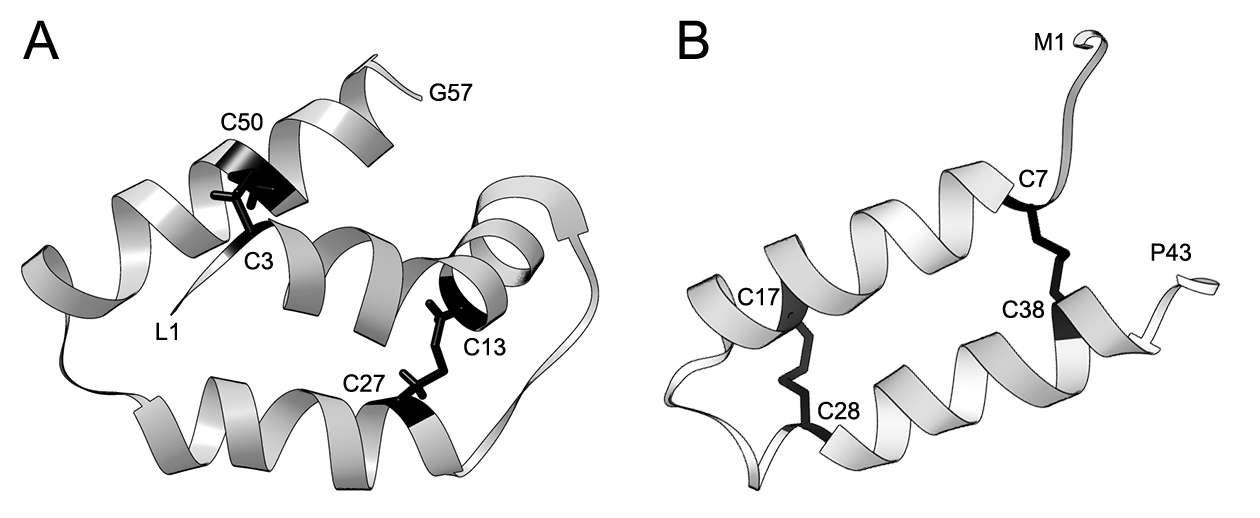

Supplement: S5 Fig — (A) Fragment of barley lipid transfer protein with a right-handed superhelical motif constrained by the enclosed disulphide bonds Cys3-Cys50/Cys13-Cys27. Note the asymmetrical cysteine spacing motif Cys-9-Cys-13-Cys-22-Cys. (B) Cysteine alpha-hairpin motif of the human p8MTCP1 protein, stapled with the enclosed disulphide bonds Cys7-Cys38/Cys17-Cys28. Note the symmetrical cysteine spacing motif Cys-9-Cys-10-Cys-9-Cys. (TIF) [file pone.0138787.s005.tif]

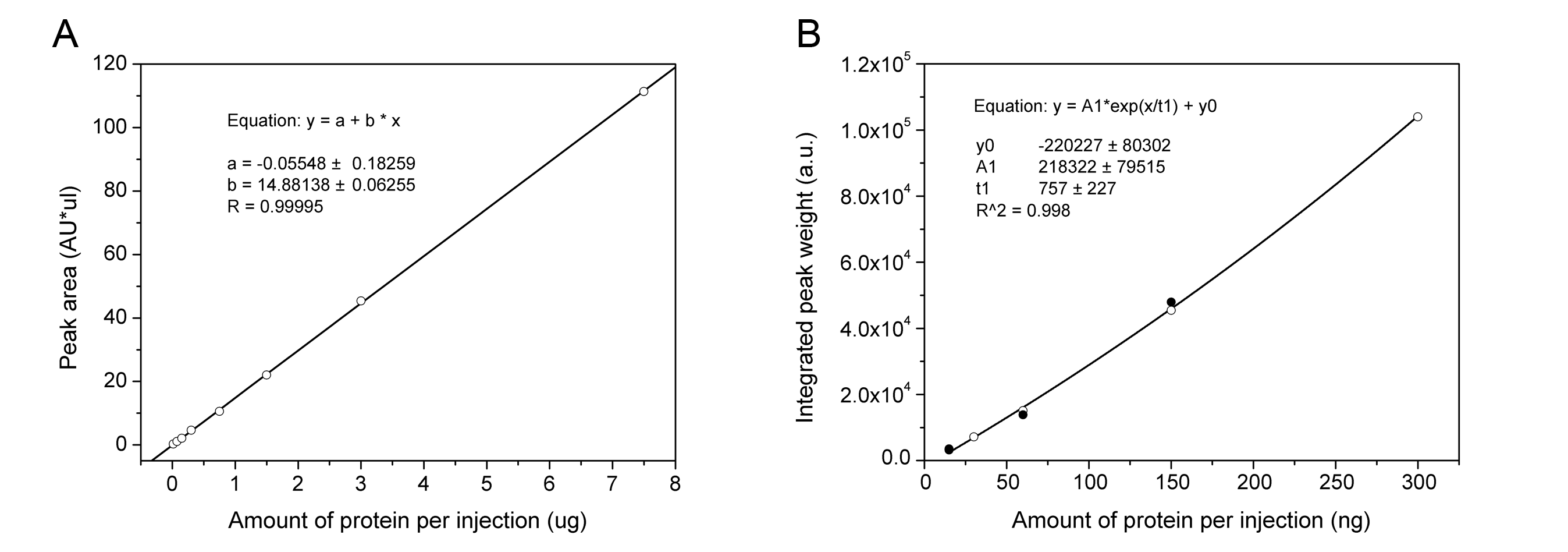

Supplement: S6 Fig — (A) Calibration plot used to estimate the amount of CRASP in fractions of perfusion fluid with HPLC. The equation was obtained from a linear fit of the peak area as a function of the CRASP amount in eight calibration samples, ranging from 0.015 μg to 7.5 μg. (B) Calibration plot used to estimate the amount of CRAS in haemolymph samples with LC-ESI MS. The equation was obtained from an exponential growth fit of the integrated intensity as a function of the CRASP amount in five calibration samples, ranging from 15 ng to 300 ng. The calibration samples were analysed before (filled circles) and after (open circles) measurement of the experimental samples. (TIF) [file pone.0138787.s006.tif]
