# Supplementary material for: Cysteine-Rich Atrial Secretory Protein from the Snail Achatina achatina: Purification and Structural Characterization
Source: PLoS One. 2015 Oct 7;10(10):e0138787. doi: 10.1371/journal.pone.0138787 (PMC4596865; doi:10.1371/journal.pone.0138787)
Supplement: S1 Table — TD touch down. (DOC) [file pone.0138787.s007.doc]

| **Usage** | **Primer** | **Target** | **Direction** | **Sequence (5**′ **- 3**′**)** | **Annealing, °C** | **Amplicon length, bp** |
| --- | --- | --- | --- | --- | --- | --- |
| 3’-RACE | Nterm_F | CRASP | Forward | CTGTGAGTACCCAGATTGYGTNTTYAC | TD 65→55 | - |
|  | Int1_F | CRASP | Forward | TCCTGCTGCTGAACATHATHGARGT | TD 65→55 | - |
|  | Int2_F | CRASP | Forward | TCATCGAGGTGCCAGAYGAYATHCA | TD 65→55 | - |
| 5’-RACE | NCS1_R | CRASP | Reverse | ACTTTGGTGGAGGATAGTAGCA | 62 | - |
|  | NCS3_R | CRASP | Reverse | TGGCAACCTCCCCCTACTTTAT | 63 | - |
| High fidelity PCR | NCS1_F | CRASP | Forward | TCTCCTGCAGCGAACTTTGA | 62 | - |
| qRT-PCR | CS1_F | CRASP | Forward | GGTTGCTCCAGAAGAGTGGA | 60 | 226 |
|  | CS4_R | CRASP | Reverse | GGCAGTTGTTCTTTTGGTCGTA | 60 |
|  | PP1_F | 60S ARP | Forward | CTCCAAGCAGATGCAGCA | 60 | 345 |
|  | PP1_R | 60S ARP | Reverse | GTTCCCTTGGTGATCTTGGT | 60 |
|  | Tub_R | α-tubulin | Reverse | GCAGCAGGCCATCTACTTGC | 60 | 120 |
|  | Tub_F | α-tubulin | Forward | TCTGCAGAAAAGGCCTACCATGA | 60 |
