# Supplementary material for: Cysteine-Rich Atrial Secretory Protein from the Snail Achatina achatina: Purification and Structural Characterization
Source: PLoS One. 2015 Oct 7;10(10):e0138787. doi: 10.1371/journal.pone.0138787 (PMC4596865; doi:10.1371/journal.pone.0138787)
Supplement: S2 Table — The Stokes radius (R S) was calculated from the experimental translational diffusion coefficient (D t (20, W)) or sedimentation coefficient (S 20, W). Protein Data Bank files used for the calculation of molecular mass and partial specific volume (ῡ 20) are shown. (f/f min) frictional ratio. References: a—[60], b—[65], c—[58], d—[62], e—[56], f—[63], g—[59], h—[57], i—[64], j—[61]. (DOC) [file pone.0138787.s008.doc]

|  | **Literature data** | | |  | **Calculated** | | | |
| --- | --- | --- | --- | --- | --- | --- | --- | --- |
| **Protein, source** | ***D*_t (20, W)_** | ***S*_20, W_** | **ref** |  | ***R*_S_** | **PDB** | ***ῡ*_20_** | ***f/f*_min_** |
|  | **10^-7^ cm^2^/s** | **10^-13^ s** |  |  | **nm** | **ID** | **ml/g** |  |
| Catalase, bovine | 4,10 |  | a |  | 5,24 | 3nwl | 0,726 | 1,28 |
|  |  | 11,1 | b |  | 5,21 | - | - | 1,27 |
| Aldolase, rabbit |  | 7,40 | c |  | 4,91 | 3tu9 | 0,737 | 1,37 |
|  | 4,63 |  | d |  | 4,64 | - | - | 1,30 |
|  |  | 7,70 | b |  | 4,72 | - | - | 1,32 |
| Serum albumin, bovine | 5,93 |  | e |  | 3,62 | 4f5s | 0,733 | 1,35 |
|  | 5,89 |  | f |  | 3,65 | - | - | 1,36 |
|  |  | 4,50 | b |  | 3,47 | - | - | 1,29 |
| Ovalbumin, chicken | 7,96 |  | g |  | 2,70 | 1ova | 0,738 | 1,16 |
|  | 7,73 |  | h |  | 2,78 | - | - | 1,20 |
|  |  | 3,40 | b |  | 2,90 | - | - | 1,25 |
| Carbonic anhydrase, bovine |  | 2,89 | i |  | 2,37 | 1v9e | 0,732 | 1,17 |
|  | 9,40 |  | j |  | 2,28 | - | - | 1,12 |
| Chymotrypsinogen A, bovine | 9,30 |  | g |  | 2,31 | 4q2k | 0,732 | 1,18 |
|  | 9,50 |  | h |  | 2,26 | - | - | 1,16 |
|  | 9,23 |  | a |  | 2,33 | - | - | 1,19 |
| Ribonuclease A, bovine | 1,07 |  | a |  | 2,01 | 1g8v | 0,712 | 1,28 |
|  | 1,16 |  | h |  | 1,85 | - | - | 1,18 |
| Bovine pancreatic | 1,49 |  | h |  | 1,44 | 4pti | 0,718 | 1,17 |
| trypsin inhibitor | 1,29 |  | g |  | 1,66 | - | - | 1,35 |
